# Phycocyanobilin biosynthesis in *Galdieria sulphuraria* requires isomerization of phycoerythrobilin synthesized by bilin reductases

**DOI:** 10.1111/febs.70391

**Published:** 2026-01-10

**Authors:** Federica Frascogna, Nathan C. Rockwell, Jana Hartmann, Julie M. Mudler, Nicole Frankenberg‐Dinkel

**Affiliations:** ^1^ Department of Microbiology RPTU University Kaiserslautern‐Landau Germany; ^2^ Department of Molecular and Cellular Biology University of California at Davis CA USA

**Keywords:** linear tetrapyrrole, phycobilisome, phycocyanobilin, pigment biosynthesis, rhodophytes

## Abstract

Phycobiliproteins are essential components of the light‐harvesting antennae in cyanobacteria and red algae, requiring covalently bound open‐chain tetrapyrrole chromophores (bilins) for proper function. In the red alga *Galdieria sulphuraria*, the primary chromophore is phycocyanobilin (PCB), despite the apparent presence of only biosynthetic genes for phycoerythrobilin (PEB) biosynthesis (*PEBA* and *PEBB*). This observation suggests the presence of an alternative, atypical biosynthetic pathway for PCB. In this study, we confirmed the presence of PEB:PCB isomerase activity in an enriched protein fraction from *G. sulphuraria*. To further investigate this unusual pathway, we combined *in silico* analyses with biochemical assays. Phylogenetic analyses confirmed the placement of the *G. sulphuraria* ferredoxin‐dependent bilin reductases within the PEBA and PEBB lineages, typically associated with PEB synthesis, whereas the related red alga *Cyanidioschyzon merolae* was found to contain only *PCYA*. This gene distribution presents a functional paradox. *G. sulphuraria* harbors PEB biosynthesis genes but no detectable PEB chromophores and lacks known PCB‐synthesizing enzymes despite containing PCB. Functional characterization of recombinant *Gs*PEBA (*G. sulphuraria* PEBA) and *Gs*PEBB (*G. sulphuraria* PEBB) confirmed their roles in PEB synthesis, demonstrating that these enzymes have not evolved to synthesize PCB or act as isomerases despite their phylogenetic placement. In contrast, *Cm*PCYA (*C. merolae* PCYA) catalyzed direct PCB formation from biliverdin. Together, these findings reveal an atypical isomerase‐based pathway for PCB biosynthesis in *G. sulphuraria*, expanding our understanding of bilin metabolism and providing new insight into the evolutionary flexibility of photosynthetic pigment biosynthesis in Rhodophyta.

## Graphical abstract

The biosynthesis of bilins, tetrapyrroles essential for light harvesting and sensing, is performed by specific enzymes (FDBRs). In *Galdieria sulphuraria*, both phycobiliprotein types bind phycocyanobilin, despite lacking the canonical synthesizing gene PCYA. Instead, PEBA and PEBB are encoded, producing phycoerythrobilin, proposed to be later isomerized to phycocyanobilin. Phylogenetic and enzymatic analyses presented in this work confirm this unique biosynthetic route, highlighting a unique evolutionary diversification of pigment biosynthesis in Rhodophytes.

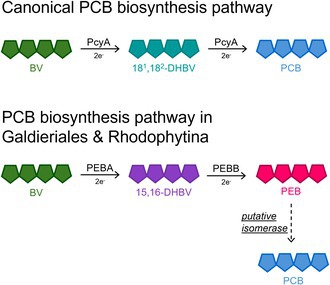

**Abbreviations**
AHT: anhydrotetracycline
APC: allophycocyanin
BSA: bovine serum albumin
BV: biliverdin IXα
*Cm*PCYA: *Cyanidioschyzon merolae* PCYA
CV: column volume
DAD: diode‐array detector
DHBV: dihydrobiliverdin
DMSO: dimethyl sulfoxide
EDTA: ethylenediaminetetraacetic acid
Fd: ferredoxin
FDBR: ferredoxin‐dependent bilin reductase
FNR: ferredoxin‐NADP^+^ reductase
fwd: forward
*Gs*PEBA: *Galdieria sulphuraria* PEBA
*Gs*PEBB: *Galdieria sulphuraria* PEBB
HEPES: 2‐[4‐(2‐hydroxyethyl)piperazin‐1‐yl]ethanesulfonic acid
HO: heme oxygenase
HPLC: high‐performance liquid chromatography
HY2: phytochromobilin synthase
IPTG: isopropyl β‐d‐1‐thiogalactopyranoside
kDa: kilodalton
LB: lysogeny broth
MW: molecular weight
MWCO: molecular weight cutoff
NADP: nicotinamide‐adenine‐dinucleotide phosphate
NRS: NADPH‐regenerating system
OD600: optical density at 600 nm
PBP: phycobiliprotein
PBS: phycobilisome
PC: phycocyanin
PCB: phycocyanobilin
PcyA: phycocyanobilin:ferredoxin oxidoreductase
PE: phycoerythrin
PEB: phycoerythrobilin
PEBA: 15,16‐dihydrobiliverdin (DHBV):ferredoxin oxidoreductase
PEBB: phycoerythrobilin:ferredoxin oxidoreductase
PEBS: phycoerythrobilin synthase
PetF: ferredoxin
PetH: ferredoxin‐NADP^+^ reductase
pH: potential hydrogen
PTFE: polytetrafluoroethylene
PUB: phycourobilin
PUBS: phycourobilin synthase
PVB: phycoviolobilin
PVDF: polyvinylidene fluoride
PΦB: phytochromobilin
rev: reverse
r.p.m.: rounds per minute
SEC: size exclusion chromatography
SH‐aLRT: Shimodaira–Hasegawa approximate likelihood ratio test
*Syc*PebA: *Synechococcus* sp. WH8020 PebA
*Syn*PcyA: *Synechocystis* sp. PCC 6803 PcyA
TBE: transfer bootstrap expectation
TES: *N*‐[tris(hydroxymethyl)methyl]‐2‐aminoethanesulfonic acid
TFA: trifluoroacetic acid
Tris: tris(hydroxymethyl)aminomethane
UF‐Boot: ultrafast bootstrap
UV–Vis: ultraviolet–visible spectrophotometry
v/v: volume per volume
w/v: weight per volume
*Z*/*E*
: z*usammen*/*entgegen*

## Introduction

Phycobiliproteins (PBPs) are a family of fluorescent proteins primarily found in cyanobacteria, red algae (Rhodophytes), glaucophytes, and cryptophytes, where they play a crucial role in light‐harvesting during photosynthesis [1, 2, 3, 4]. PBPs enhance the light‐harvesting machinery of these organisms by absorbing light in the 500–650 nm range, the so‐called “Green gap” where chlorophyll poorly absorbs [5, 6, 7, 8]. These proteins are most often assembled into large complexes known as phycobilisomes (PBSs), which transfer the captured light energy to both photosystem II and photosystem I reaction centers [9, 10, 11, 12]. The ability of PBPs to harvest light is dependent on open‐chain tetrapyrrole chromophores, called phycobilins (or short bilins). The early branching Rhodophytes of the Cyanidiales served as model organisms to study the biosynthesis of these chromophores. The first studies on *Galdieria sulphuraria* (formerly mistaken for *Cyanidium caldarium*: [13, 14, 15, 16, 17]) implied that the open‐chain tetrapyrrole biliverdin IXα (BV) is the precursor of all phycobilins [18, 19]. In subsequent studies by Beale, Cornejo, and colleagues, BV was shown to be reduced by an unknown ferredoxin‐dependent enzyme to phycoerythrobilin (PEB) and then converted, via isomerization, to phycocyanobilin (PCB) [20, 21, 22]. Around a decade later, the enzymes responsible for BV reduction were identified as the so‐called ferredoxin‐dependent bilin reductases (FDBRs) [23, 24]. In general, the first universal step in the biosynthesis of bilins is the ferredoxin‐dependent oxidation of heme at the α‐methine bridge by heme oxygenase (HO) [25, 26, 27]. This reaction yields BV and additionally liberates H_2_O, CO, and Fe^2+^ [28, 29]. BV is subsequently reduced to specific bilins by FDBRs (Fig. S1) [24, 30, 31, 32, 33, 34, 35, 36, 37, 38, 39, 40, 41]. FDBRs are a conserved family of enzymes that are ubiquitous in oxyphototrophs [42] with varying in substrate specificity and reaction regiospecificity, allowing the production of different bilins. Although Beale and Cornejo showed that PCB production occurs via PEB in *G. sulphuraria*, an enzyme directly reducing BV to PCB was subsequently discovered in cyanobacteria [24, 33]. In cyanobacteria, some other bacteria, glaucophyte algae, and a small subset of cryptophyte algae, this phycocyanobilin oxidoreductase (PcyA) is responsible for the 4‐electron reduction of BV to PCB via 18^1^,18^2^‐dihydrobiliverdin (18^1^,18^2^‐DHBV) [33, 43, 44, 45, 46]. More recently, PCB was found to be synthesized in a similar fashion by the FDBR HY2 of streptophyte algae, with the possible occurrence of either 18^1^,18^2^‐DHBV or phytochromobilin (PФB) as the biosynthetic intermediate [31, 32]. However, in these organisms, PCB is bound to phytochromes and used for light sensing rather than light harvesting [32, 47]. Spectroscopic analyses have suggested that the PBSs of *Galdieria* species, like those of other Cyanidiales, consist solely of an allophycocyanin (APC) core and peripheral rods containing phycocyanin (PC), both of which bind PCB chromophores [48, 49, 50, 51, 52]. Interestingly, while *Cyanidioschyzon merolae* possesses a *PCYA* gene, *G. sulphuraria* and related species lack *PCYA* but instead harbor *PEBA* and *PEBB* genes [42]. This presents a biochemical paradox: *G. sulphuraria* appears to require PCB‐binding PBPs yet lacks canonical PCB biosynthesis genes; it contains PEB biosynthesis genes (*PEBA* and *PEBB*), even though no PEB chromophores have been detected in its PBSs and are instead associated with the PBSs of later‐branching rhodophytes.

To address this conundrum, we combined *in silico* approaches with enzymatic assays. *In vivo* experiments first confirmed the presence of PEB:PCB isomerase activity in protein extracts [21]. Next, phylogenetic analyses of FDBR sequences from Rhodophytes were conducted to determine their evolutionary placement and to make functional inferences. These analyses reliably grouped *G. sulphuraria* and *C. merolae* FDBRs into the expected clades. However, it is well established that phylogenetic proximity alone provides limited predictive power regarding catalytic specificity in FDBRs. Even with conserved active‐site residues, precise enzyme function cannot always be inferred from sequence data alone [31, 37, 39]. We therefore performed functional assays using recombinant proteins. These results confirmed that *G. sulphuraria* contains authentic PEBA (*Gs*PEBA) and PEBB (*Gs*PEBB) enzymes, whereas *C. merolae* instead contains an authentic PCYA (*Cm*PCYA). Our results support the earlier isomerase hypothesis of Beale and Cornejo [21] for PCB biosynthesis in *G. sulphuraria*. This atypical biosynthetic route not only resolves the apparent paradox but also offers broader insights into the evolution of photosynthetic pigment pathways originating from primary endosymbiosis in the Archaeplastida supergroup.

## Results

### Isomerization of PEB to PCB

**Fig. 1:**
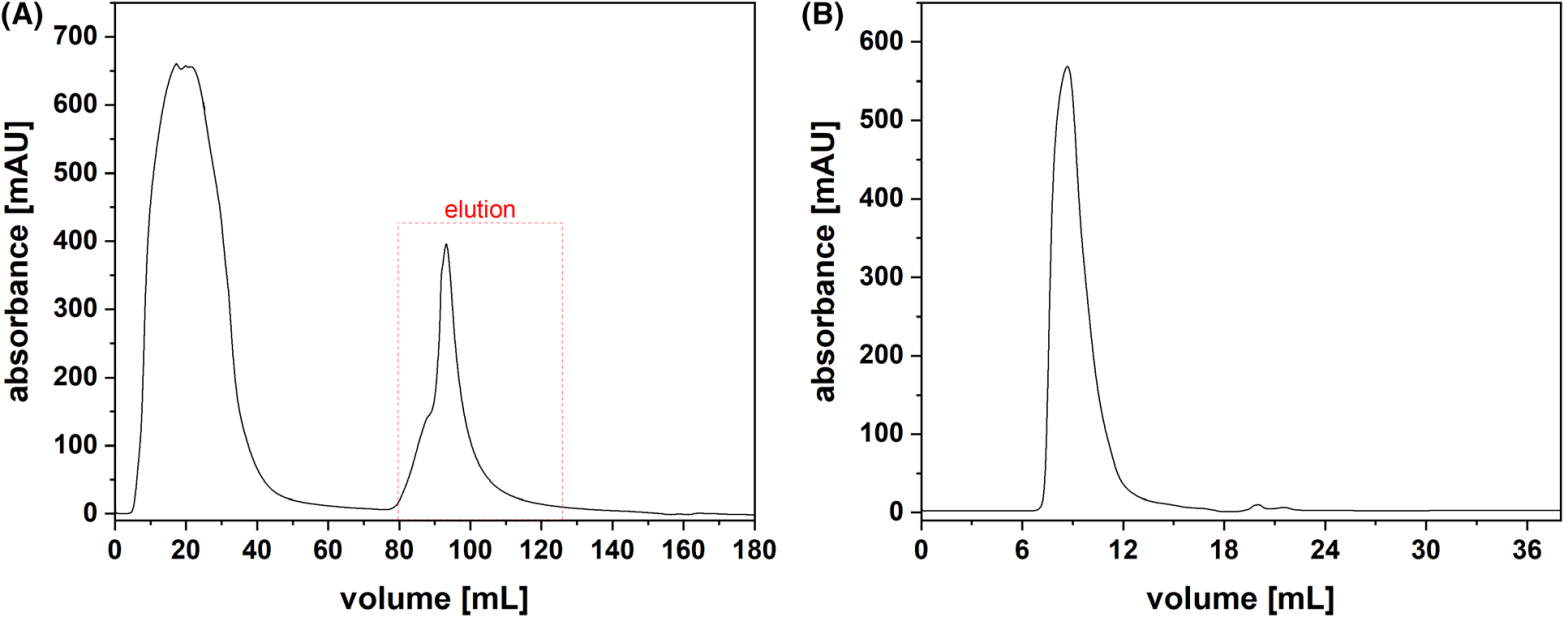
Affinity chromatography and size exclusion chromatography of a protein‐enriched fraction from *Galdieria sulphuraria*. (A) Affinity chromatography (*n* = 3) was performed using the ÄKTA™ pure 25 purification system equipped with a column packed with Blue Sepharose™ 6 Fast Flow. Equilibration and washing with ‘Assay buffer’ were followed by elution with ‘Elution buffer’. Absorbance was continuously measured at 280 nm. (B) Size exclusion chromatography (*n* = 3) was performed using the ÄKTA™ pure 25 purification system equipped with a Superdex™ 75 10/300 GL, equilibrated with ‘Assay buffer’. Absorbance was continuously measured at 280 nm.

In the present study, we aimed to elucidate the atypical biosynthetic pathway of PCB in *G. sulphuraria*. To this end, our first goal was to confirm the PEB:PCB isomerase activity that was described more than 30 years ago [21]. Following the mechanical disruption of the *G. sulphuraria* cells, two ammonium sulfate precipitations were carried out. After dialysis of the precipitated protein fraction from the second ammonium sulfate fractionation, Blue Sepharose affinity chromatography was performed (Fig. 1A). The column material interacted with target proteins in the algal lysate, eluting at ~ 90 mL (Fig. 1A). This fraction was subsequently treated with a 75% saturating concentration of ammonium sulfate. Precipitated proteins were resuspended in assay buffer and subjected to size exclusion chromatography (Fig. 1B). The previous findings of Beale and Cornejo indicated that an isolated fraction with a molecular mass > 60 kDa exhibited isomerase activity. Using the elution profile of albumin as reference (66 kDa, Fig. S2), the fractions eluting between 7 and 12 mL were collected and employed in an isomerase activity assay. These fractions all displayed a bluish color, consistent with the possible presence of endogenous PCB but not that of the pink‐colored PEB.

**Fig. 2:**
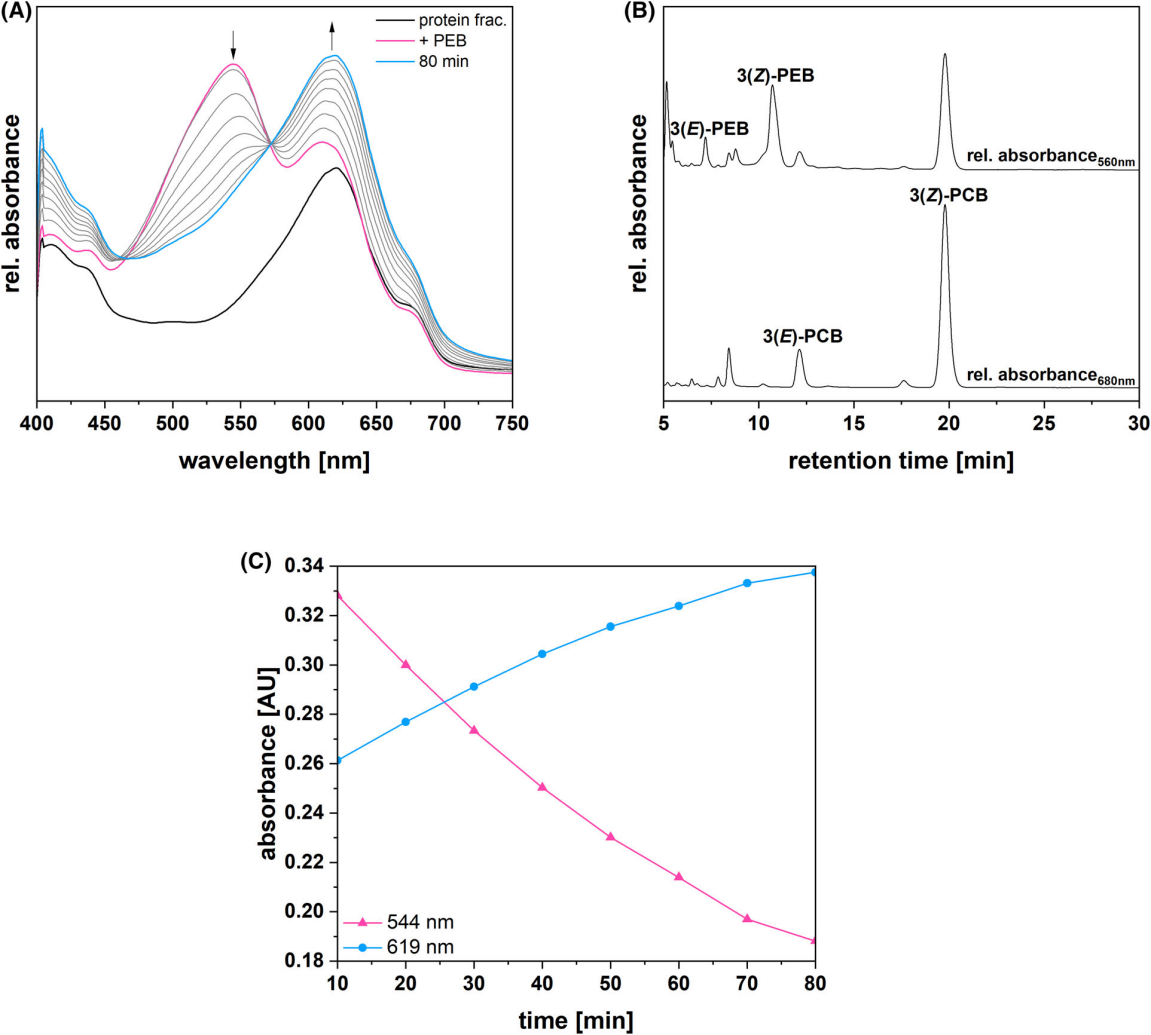
Isomerase activity assay of a *Galdieria sulphuraria* enriched protein fraction, identification of products and kinetics of PEB decay/PCB appearance. (A) Isomerase activity assay of a *G. sulphuraria* enriched protein fraction (*n* = 3). The solid black line represents the absorbance of the protein fraction (protein frac.). The enriched protein fraction was incubated with 14 μm PEB at 30 °C. The pink spectrum was acquired immediately after the addition of PEB (+PEB). The course of absorbance, indicated by the arrows, was recorded every 10 min for 80 min. The cyan spectrum represents the end spectrum, recorded at 80 min (80 min). A 10‐point Savitzky–Golay filter was applied to smooth the curves. (B) HPLC analysis of the isomerase assay products (*n* = 3). The products were separated on a reversed‐phase 5 μm C18 Luna column (Phenomenex), with a mobile phase consisting of 50%_v/v_ acetone and 50%_v/v_ 20 mm formic acid, at a flow rate of 0.6 mL·min^−1^. Absorbance was monitored continuously at 560 and 680 nm. The upper trace indicates the chromatogram recorded at the constant DAD wavelength of 560 nm (rel. absorbance_560nm_). The lower trace indicates the chromatogram recorded at the constant DAD wavelength of 680 nm (rel. absorbance_680nm_). 3(*E*)‐PCB, 3(*E*)‐phycocyanobilin; 3(*E*)‐PEB, 3(*E*)‐phycoerythrobilin; 3(*Z*)‐PCB, 3(*Z*)‐phycocyanobilin; 3(*Z*)‐PEB, 3(*Z*)‐phycoerythrobilin. (C) The kinetics of PEB decay and PCB appearance from panel A were fit to single exponential functions. The decay of PEB (monitored at 544 nm: filled pink triangles; *k*_app_ = 0.014 ± 0.001 min^−1^, *r* ^2^ = 0.9992) and rise of PCB (monitored at 619 nm: filled cyan circles; *k*_app_ = 0.015 ± 0.001 min^−1^, *r* ^2^ = 0.9994) occurred at equivalent rates.

About 14 μm purified PEB was added to 1.5 mL concentrated protein fraction, and potential conversion to PCB was monitored via spectroscopy at 30 °C over the course of 80 min (Fig. 2A). The protein fraction initially showed an absorption peak at 619 nm, consistent with the presence of PC, and shoulders at 410, 440, and 680 nm representing chlorophyll *a* (Fig. 2A: protein frac.). Addition of pure PEB resulted in the formation of a new peak at 544 nm (Fig. 2A: +PEB). At the final time point, the UV–Vis spectrum displayed apparently complete conversion of the 544 nm peak to PCB, represented by the 619 nm peak (Fig. 2A: 80 min). HPLC analysis of the 80 min assay sample revealed the presence of PCB and remaining amounts of PEB (Fig. 2B), whereas the double peak eluting at ~ 8.5 min and absorbing at 560 nm could indicate the presence of 15,16‐dihydrobiliverdin (DHBV). PEB decay and PCB appearance were found to occur at equivalent rates (Fig. 2C).

**Fig. 3:**
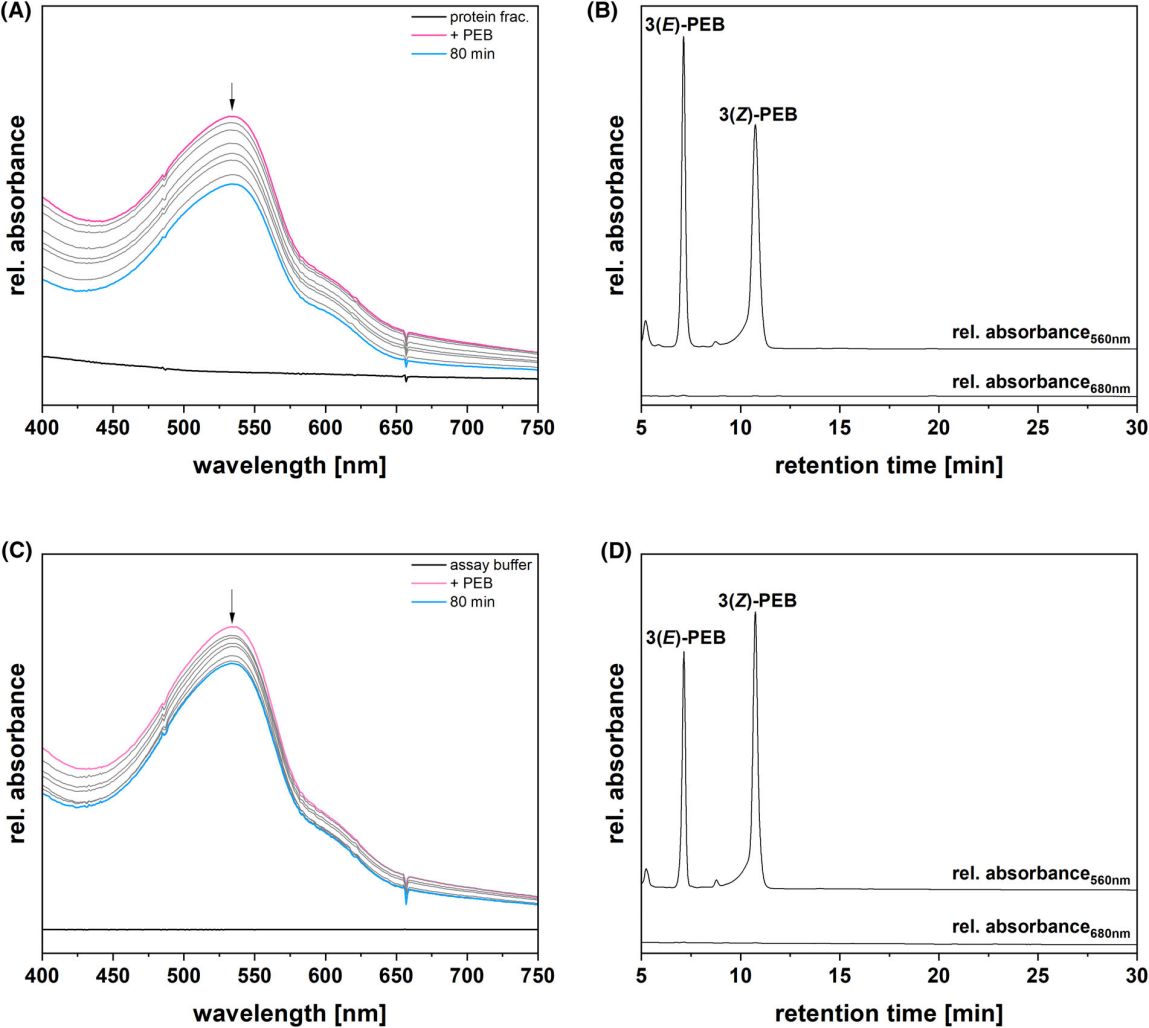
Negative controls of isomerase activity assay and identification of products. (A) Isomerase activity assay of a heat‐inactivated *Galdieria sulphuraria* enriched protein fraction (*n* = 3). The solid black line represents the absorbance of the protein fraction subjected to heat‐inactivation at 95 °C for 10 min (protein frac.). The enriched protein fraction was incubated with 14 μm PEB at 30 °C. The pink spectrum was acquired immediately after the addition of PEB (+PEB). The course of absorbance, indicated by the arrow, was recorded every 10 min for 80 min. The cyan spectrum represents the end spectrum, recorded at 80 min (80 min). (B) HPLC analysis of the isomerase assay products (*n* = 1, as one analysis was enough to unequivocally identify the products). The products were separated on a reversed‐phase 5 μm C18 Luna column (Phenomenex), with a mobile phase consisting of 50%_v/v_ acetone and 50%_v/v_ 20 mm formic acid, at a flow rate of 0.6 mL·min^−1^. Absorbance was monitored continuously at 560 and 680 nm. The upper trace indicates the chromatogram recorded at the constant DAD wavelength of 560 nm (rel. absorbance_560nm_). The lower trace indicates the chromatogram recorded at the constant DAD wavelength of 680 nm (rel. absorbance_680nm_). 3(*E*)‐PEB, 3(*E*)‐phycoerythrobilin; 3(*Z*)‐PEB, 3(*Z*)‐phycoerythrobilin. (C) Isomerase activity assay performed substituting the *G. sulphuraria* enriched protein fraction with ‘Assay buffer’ (*n* = 3). The solid black line represents the absorbance of the ‘Assay buffer’ (assay buffer). The ‘Assay buffer’ was incubated with 14 μm PEB at 30 °C. The pink spectrum was acquired immediately after the addition of PEB (+PEB). The course of absorbance, indicated by the arrow, was recorded every 10 min for 80 min. The cyan spectrum represents the end spectrum, recorded at 80 min (80 min). A 10‐point Savitzky–Golay filter was applied to smooth the curves. (D) HPLC analysis of the isomerase assay products (*n* = 1, as one analysis was enough to unequivocally identify the products). The products were separated on a reversed‐phase 5 μm C18 Luna column (Phenomenex), with a mobile phase consisting of 50%_v/v_ acetone and 50%_v/v_ 20 mm formic acid, at a flow rate of 0.6 mL·min^−1^. Absorbance was monitored continuously at 560 and 680 nm. The upper trace indicates the chromatogram recorded at the constant DAD wavelength of 560 nm (rel. absorbance_560nm_). The lower trace indicates the chromatogram recorded at the constant DAD wavelength of 680 nm (rel. absorbance_680nm_). 3(*E*)‐PEB, 3(*E*)‐phycoerythrobilin; 3(*Z*)‐PEB, 3(*Z*)‐phycoerythrobilin.

The dependence of this activity on an enzymatic factor was verified via negative controls (Fig. 3). In the first control, the enriched protein fraction was heat‐inactivated before the assay (Fig. 3A,B). In the second control, a buffer blank was used instead of the protein fraction (Fig. 3C,D). None of these control assays exhibited detectable isomerase activity, consistent with the original conclusion that this reaction is dependent on an enzymatic component.

### Phylogenetic analysis of FDBRs from *G. sulphuraria* and *C. merolae*

These results thus confirm the presence of a PEB:PCB isomerase activity as previously seen by Beale and Cornejo [21], suggesting the presence of a dedicated PEB‐to‐PCB isomerase in *Galdieria* spp. However, subsequent identification of FDBRs has shown that they can carry out reactions inconsistent with their phylogenetic placement [24, 32, 42]. Moreover, additional FDBR lineages are still being discovered [32]. It thus seemed plausible that the earlier assessment of FDBRs in *G. sulphuraria* was incomplete, in that the earlier phylogenetic analyses of *Galdieria* FDBRs were incorrect, or that the enzymatic activities of those FDBRs might not match expected ones based on phylogenetic analysis. There are three known lineages of FDBRs [24, 42]. Members of the PebA/PebS/PUBS lineage utilize BV as substrate and carry out reduction of the 15,16‐double bond in a 2‐electron reduction to generate 15,16‐dihydrobiliverdin (15,16‐DHBV). PUBS carries out an additional reduction of the 4,5‐double bond, resulting in a 4‐electron reduction [38, 39], and is found in some members of the Viridiplantae (prasinophyte/chlorophyte algae, streptophyte algae, and land plants [53]). PebS instead carries out an additional reduction of the bilin A‐ring to generate PEB as a final product in a different 4‐electron reduction and is found in some cyanophages [36, 54]. Members of the PebB/HY2 lineage carry out the same A‐ring reduction without reduction of the 15,16‐double bond. PebB and HY2 differ in substrate specificity: PebB utilizes 15,16‐DHBV and synthesizes PEB, whereas HY2 utilizes BV as substrate and is found in streptophytes [24, 30]. HY2 enzymes from streptophyte algae also carry out an additional reduction of the BV 18‐vinyl moiety, resulting in synthesis of PCB, whereas those from land plants do not perform this reduction and generate PΦB [30, 31]. The third FDBR lineage comprises PcyA, PcyX, and the recently described pre‐PcyA proteins [24, 32]. PcyA carries out reduction of the 18‐vinyl moiety and BV A‐ring to generate PCB, effectively performing the same reactions as algal HY2 proteins [33]. PcyA sequences are rarely found in rhodophyte and cryptophyte algae but are ubiquitous in known prasinophyte/chlorophyte algae and in cyanobacteria [42]. PcyA is also found in some other bacteria, such as *Bradyrhizobium* sp. ORS278 [37, 55]; such sequences are phylogenetically distinct from other PcyA proteins and form a clade with PcyX (the AX clade; [32]). Pre‐PcyA proteins form the early branches within this clade and carry out the reduction of the A‐ring, with some such proteins also carrying out subsequent reduction of the C15=C16 [32]. These proteins thus synthesize PΦB and PEB and are found in diverse heterotrophic and anoxygenic photosynthetic bacteria. PEB is also synthesized by PcyX, which is found in some phage genomes but not in cyanophages [37, 56].

Structures have been solved for cyanobacterial PcyA and PebA [44, 45, 57], phage PebS and PcyX [37, 54], cryptophyte PEBB [58], and tomato HY2 [59]. All structures reveal a conserved fold with a central beta sheet and a conserved binding pocket for bilin substrates. The FDBR fold is also seen in two other known enzyme families, red chlorophyll catabolite reductase (RCCR) and oxygen‐dependent coproporphyrinogen oxidase (CPO), both tetrapyrrole‐binding proteins [44, 60]. FDBRs do not share significant sequence homology with CPO sequences, making this shared overall fold surprising. Homology to RCCR can be detected in sequence searches, and RCCR has been used as an outgroup for phylogenetic analysis of FDBRs [24, 32]. Homology between different FDBRs can usually be detected, but some lineages can be quite distant. Given the recent identification of additional FDBR lineages, we re‐examined the *G. sulphuraria* genome to test for the presence of additional FDBR homologs using both blast and hmmer searches (see Materials and methods). A broad panel of known or candidate FDBRs was used as queries, along with Arabidopsis RCCR and a candidate bacterial CPO. These searches detected only three *G. sulphuraria* sequences (Table S1): EME26592, annotated as PEBB; EME26797, annotated as PEBA; and EME30735, annotated as CPO. We therefore conclude that there is not a previously undetected PcyA homolog in *G. sulphuraria*.

It still seemed plausible that previous phylogenetic placements were incorrect or that the FDBRs in *G. sulphuraria* did not perform the reactions expected based on those placements. Notably, several routes exist for synthesis of PEB even among the small set of enzymes that have been experimentally characterized to date. In cyanobacteria, the biosynthesis of PEB begins with a 2‐electron reduction of BV to 15,16‐DHBV catalyzed by PebA, followed by a second 2‐electron reduction of the A‐ring diene system by PebB to produce PEB [24]. Cryptophyte algae possess PEBA and PEBB sequences, and PEBB from *Guillardia theta* has the same substrate specificity and reaction regiospecificity as its cyanobacterial orthologs [58, 61]. However, there are other routes to synthesize PEB. The pre‐PcyA enzyme MBL9008304 can synthesize PEB from BV in a 4‐electron reduction via an apparent PΦB intermediate [32], whereas the phage enzymes PebS and PcyX can both synthesize PEB in 4‐electron reductions via 15,16‐DHBV and thereby mimic the cyanobacterial pathway with a single enzyme [36, 37, 54, 56]. PebS and PcyX have been classified within the PebA and PcyA lineages, respectively, highlighting the occasionally weak correlation between phylogenetic analysis and regiospecificity in FDBRs. Moreover, structure/function relationships in FDBRs are not well understood or reliably predictable. For example, the streptophyte algal FDBR *Kfla*HY2 normally carries out a 4‐electron reduction of BV to PCB, but site‐directed mutagenesis demonstrated that the final product could be changed from PCB to PEB with only one amino acid substitution [31, 32]. We therefore chose to update the phylogenetic analysis of rhodophyte FDBRs and to establish the properties of those proteins *in vitro* as a means of testing the need for an isomerase as opposed to unanticipated behavior of known FDBR sequences.

**Fig. 4:**
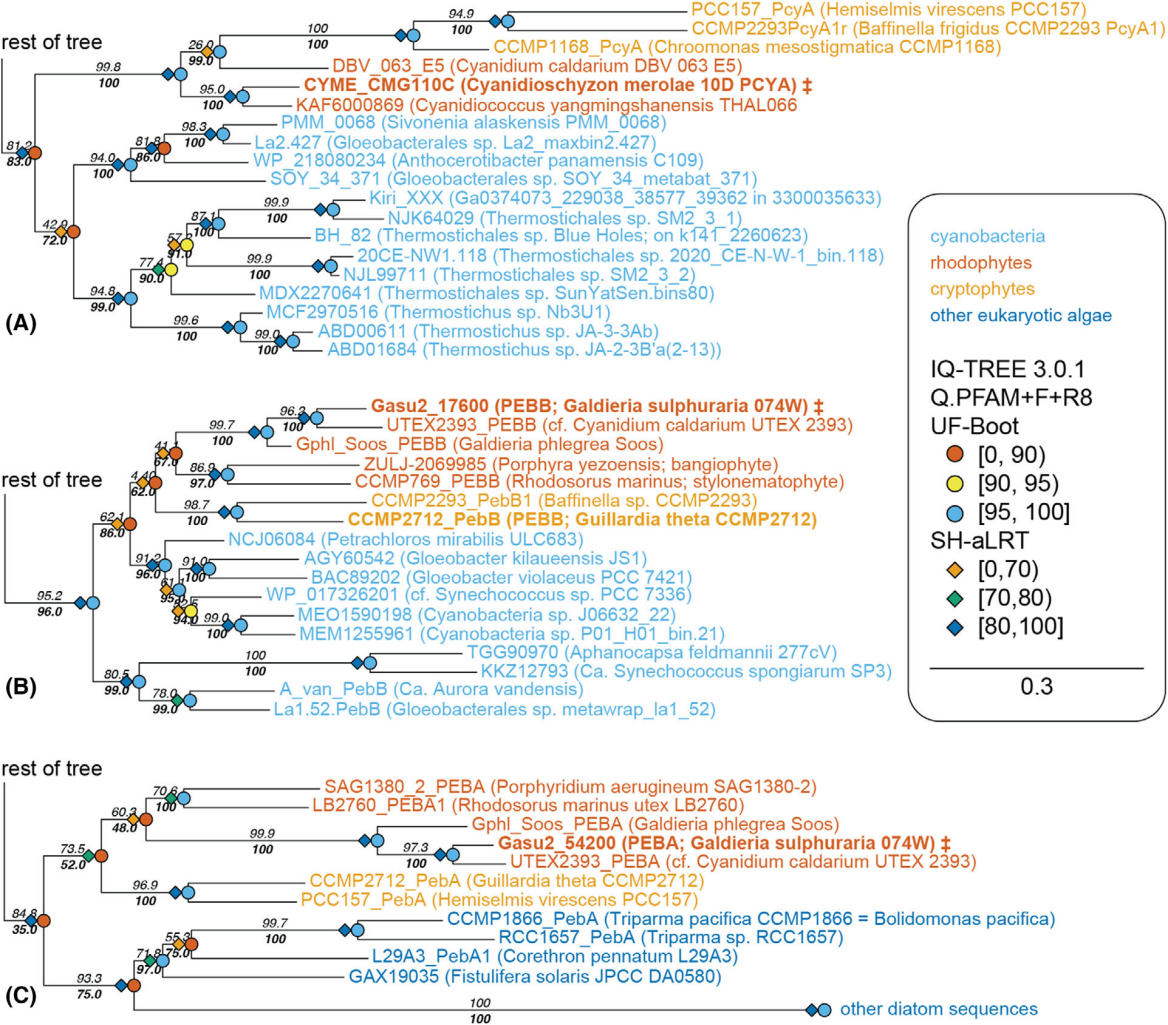
Phylogenetic analysis of FDBRs. Detailed views of the phylogeny inferred using iq‐tree version 3.0.1 are shown for the regions including *Cm*PCYA (A), *Gs*PEBB (B), and *Gs*PEBA (C). Taxa are color‐coded as indicated, and characterized sequences are indicated in bold. The full, annotated tree is presented in Fig. S4 and is compared to the tree inferred using phyml in Fig. S3. Supports are indicated symbolically; explicit SH‐aLRT supports are in italics, and ufboot supports are in bold italics. ‡, sequences examined in this manuscript. The original alignment, gap‐trimmed input file, and output files are available via DataDryad (DataDryad: https://doi.org/10.5061/dryad.hhmgqnksx).

We began with an updated phylogenetic analysis building on the recent identification of pre‐PcyA proteins [32]. We conducted additional searches for additional candidate FDBR sequences and for sequences related both to RCCR and to the much more distantly related CPO, with an emphasis on metagenomic analyses that have appeared after the previous work [62, 63, 64, 65]. This allowed the use of CPO as an outgroup in the current analysis, which seemed preferable given that it is a much more widespread enzyme than RCCR. We then carried out two maximum‐likelihood phylogenetic analyses using different software, substitution models, and search algorithms. In one case, we used phyml‐3.3 with the WAG model and SPR algorithm [66]; this analysis was similar to that used in the analysis of pre‐PcyA proteins [32] that used the earlier version 3.1. This approach used 100 non‐parametric bootstraps, with supports evaluated using the transfer bootstrap expectation (TBE, [67]). The second analysis used iq‐tree version 3.0.1 (https://iqtree.github.io/) with the default search algorithm and with automatic choice of the substitution model via modelfinder [68], resulting in the use of the Q.PFAM+F+R8 substitution model. Supports were evaluated using the ultrafast bootstrap approximation (ufboot, [69]) and the Shimodaira–Hasegawa approximate likelihood ratio test (SH‐aLRT, [66]). Trees were then processed and visualized using treeviewer [70]. Overall, we observed good agreement between the two trees (Fig. S3). Two clades of protein sequences were placed in different positions in the two. Neither group had been identified at the time of the previous analysis [32], and neither lineage contains any characterized examples. The two phylogenies also differed in the relationship of pre‐2 and pre‐3 proteins, with phyml placing these two lineages as sister to each other as previously observed [32] and iq‐tree instead placing pre‐2 as branching before pre‐3 (Fig. S3). The agreement between the two trees was excellent for the FDBR sequences from *G. sulphuraria* and *C. merolae* (Fig. 4) and placed *Gs*PEBA, *Gs*PEBB, and *Cm*PCYA in the expected PebA, PebB, and PcyA clades, respectively. Consistent with previous analyses [32, 42], the few known cryptophyte PCYA sequences were recovered as part of a small clade also including the few known rhodophyte sequences (Fig. 4A and Fig. S3). Rhodophyte and cryptophyte PEBB sequences also formed a clade apparently descended from the plastid ancestor (Fig. 4B), with the previously noted exception of PEBB sequences from the cryptophyte genus *Hemiselmis* (Fig. S4; [42]). Rhodophyte and cryptophyte PEBA sequences also formed a clade (Fig. 4C), but in this case the clade was less well‐supported statistically. These sequences were recovered as sister to sequences from diatoms and Bolidophyceae, members of the photosynthetic ochrophyte lineage of stramenopiles that possess secondary plastids derived from red algae [71]. Cyanobacterial PebA sequences and cyanophage PebS sequences were earlier branches in this clade (Fig. S4). These analyses demonstrate that the previous assignments of *Gs*PEBA, *Gs*PEBB, and *Cm*PCYA remain valid despite the identification of additional FDBRs and reaffirm the expectation that the bilin biosynthesis pathway in *G. sulphuraria* would give rise to PEB, whereas that in *C. merolae* would instead give rise to PCB.

### Biochemical characterization of FDBRs from early branching rhodophytes

We next considered the possibility that FDBRs from *Galdieria* spp. (and those from other rhodophytes lacking PCYA) might have alternative regiospecificities allowing synthesis of PCB. Such changes in reaction products are not unknown. For example, the phage enzyme PcyX evolved from bacterial PcyA enzymes but catalyzes the synthesis of PEB rather than PCB [37]. Likewise, HY2 from streptophyte algae synthesizes PCB even though HY2 from land plants instead synthesizes PΦB; this seemed particularly relevant, given the relatively close relationship between PebB/PEBB proteins and HY2 proteins [24, 42] (also see Fig. S4). We therefore characterized the *in vitro* behavior of three FDBRs: *Gs*PEBA and *Gs*PEBB from *G. sulphuraria*, and *Cm*PCYA from *C. merolae*.

**Fig. 5:**
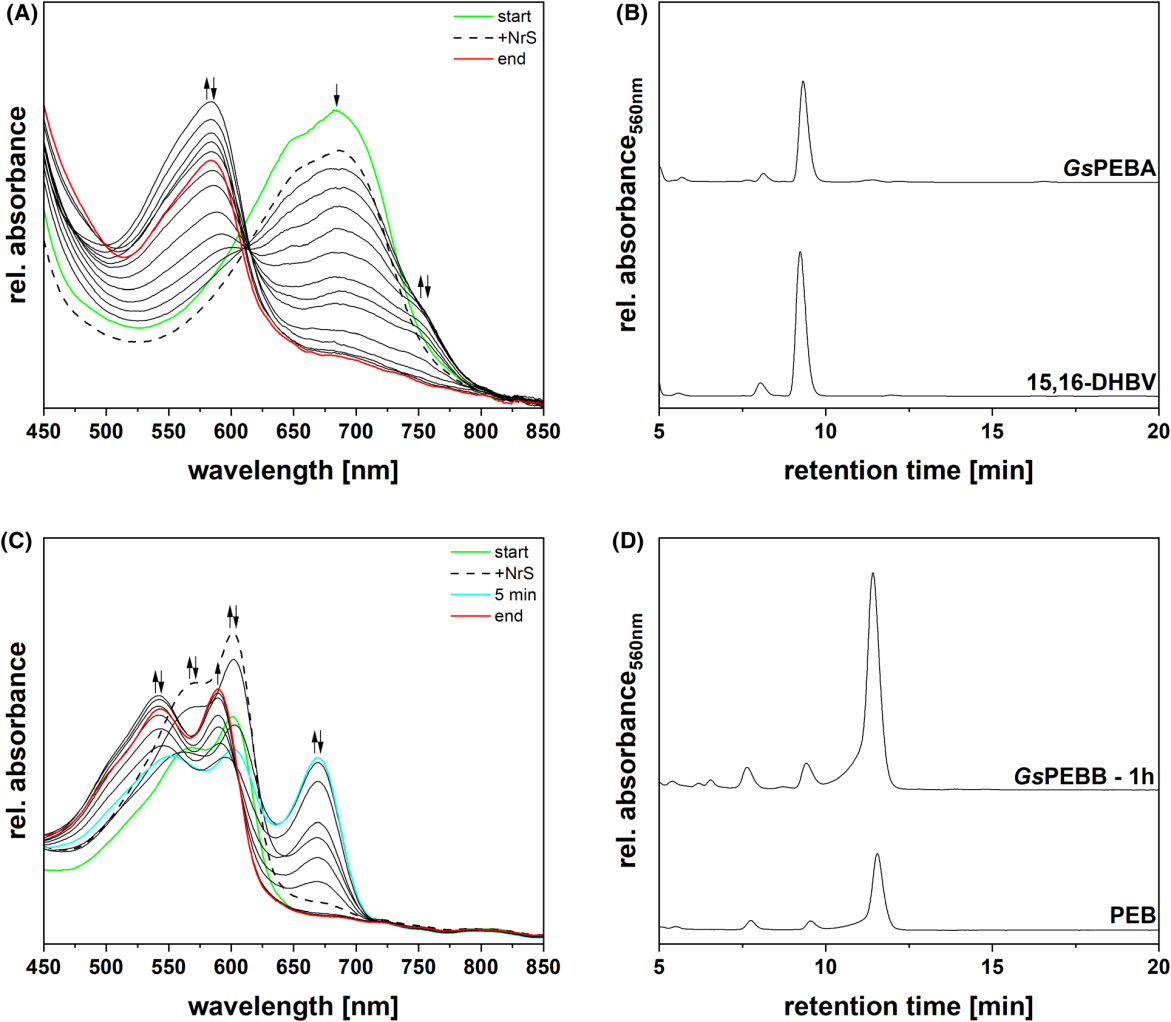
Investigation of the activity of recombinant *Gs*PEBA and *Gs*PEBB and identification of reaction products. (A) Spectra of an anaerobic bilin reductase activity assay using recombinant *Gs*PEBA and BV as the substrate (*n* = 3). The total reaction time was 20 min, with spectra recorded at 30 s intervals. For ease of understanding, only the most relevant spectra are displayed. The arrows indicate the progression of absorbance during the reaction. The green spectrum corresponds to the ‘binding spectrum’, recorded upon incubation of BV with *Gs*PEBA. The dashed line represents the first spectrum recorded after initiating the reaction via the addition of the NrS (NADPH‐regenerating system). The spectra recorded during the reaction are shown as solid black lines, while the final spectrum is colored in red. A 41‐point Savitzky–Golay filter was applied to smooth the curves. (B) HPLC analysis of the reaction products (*Gs*PEBA) (*n* = 1, as one analysis was enough to unequivocally identify the products). The products were separated on a reversed‐phase 5 μm C18 Luna column (Phenomenex), with a mobile phase consisting of 50% acetone (v/v) and 50% 20 mm formic acid (v/v), at a flow rate of 0.6 mL·min^−1^. Absorbance was monitored continuously at 560 nm. 15,16‐DHBV refers to the 15,16‐dihydrobiliverdin standard. (C) Spectra of an anaerobic bilin reductase activity assay using recombinant *Gs*PEBB and 15,16‐DHBV as the substrate (*n* = 3). The total reaction time was 1 h, with spectra recorded at 30 s intervals. For ease of understanding, only the most relevant spectra are displayed. The arrows indicate the progression of absorbance during the reaction. The green spectrum corresponds to the ‘binding spectrum’, recorded upon incubation of 15,16‐DHBV with *Gs*PEBB. The dashed line represents the first spectrum recorded after initiating the reaction via the addition of the NrS. The spectra recorded during the reaction are shown as solid black lines; the cyan spectrum represents the product formed after 5 min, while the final spectrum is colored in red. A 41‐point Savitzky–Golay filter was applied to smooth the curves. (D) HPLC analysis of the reaction products (*Gs*PEBB – 1 h) (*n* = 1, as one analysis was enough to unequivocally identify the products). The products were separated on a reversed‐phase 5 μm C18 Luna column (Phenomenex), with a mobile phase consisting of 50% acetone (v/v) and 50% 20 mm formic acid (v/v), at a flow rate of 0.6 mL·min^−1^. Absorbance was monitored continuously at 560 nm. PEB refers to the phycoerythrobilin standard.

We began by characterizing the reaction of His‐*Gs*PEBA and BV, its expected substrate, in equimolar amounts (Fig. 5A). The reductase and the substrate formed a complex with an absorbance maximum at ~ 685 nm (Fig. 5A: start). Following the addition of the NADPH‐regenerating system (NrS), used to generate a pool of reduced ferredoxin, a modest decrease in absorbance of the complex peak could be observed (Fig. 5A: +NrS). Ultimately, *Gs*PEBA revealed activity, with the ~ 585 nm absorbing product being accumulated within 12 min and subsequently undergoing nonspecific degradation over time (Fig. 5A: end). HPLC analysis confirmed *Gs*PEBA is indeed catalyzing the reduction of BV to 15,16‐DHBV, making it a canonical PebA (Fig. 5B: *Gs*PEBA).

We next examined *Gs*PEBB, which would be expected to catalyze conversion of 15,16‐DHBV to PEB. 15,16‐DHBV binding to *Gs*PEBB was demonstrated by a double absorbance peak at ~ 565 and ~ 600 nm (Fig. 5C: start). Upon initiating the reaction, a slight increase in the absorbance of both peaks was noted, attributed to residual *Gs*PEBB:15,16‐DHBV in the NrS‐injection syringe (Fig. 5C: +NrS). Within 5 min from the start, in addition to a decrease in the absorbance of the initial complex, a new peak at ~ 670 nm emerged (Fig. 5C: 5 min). Over time, the absorbance of these peaks decreased, ultimately resulting in a product absorbing at ~ 545 and ~ 590 nm (Fig. 5C: end). HPLC analysis of the final product confirmed the presence of predominantly 3(*Z*)‐PEB, alongside minor traces of 3(*E*)‐PEB and residual 15,16‐DHBV (9.4 min product). These results demonstrate the expected activity for *Gs*PEBB and rule out any isomerizing activity of *Gs*PEBB (Fig. 5D: *Gs*PEBB – 1 h). Moreover, an additional *Gs*PEBB assay was carried out using BV as the substrate. This assay confirmed that this reductase, like all other PebBs, exclusively accommodates 15,16‐DHBV and does not exhibit broad substrate specificity (Fig. S5).

**Fig. 6:**
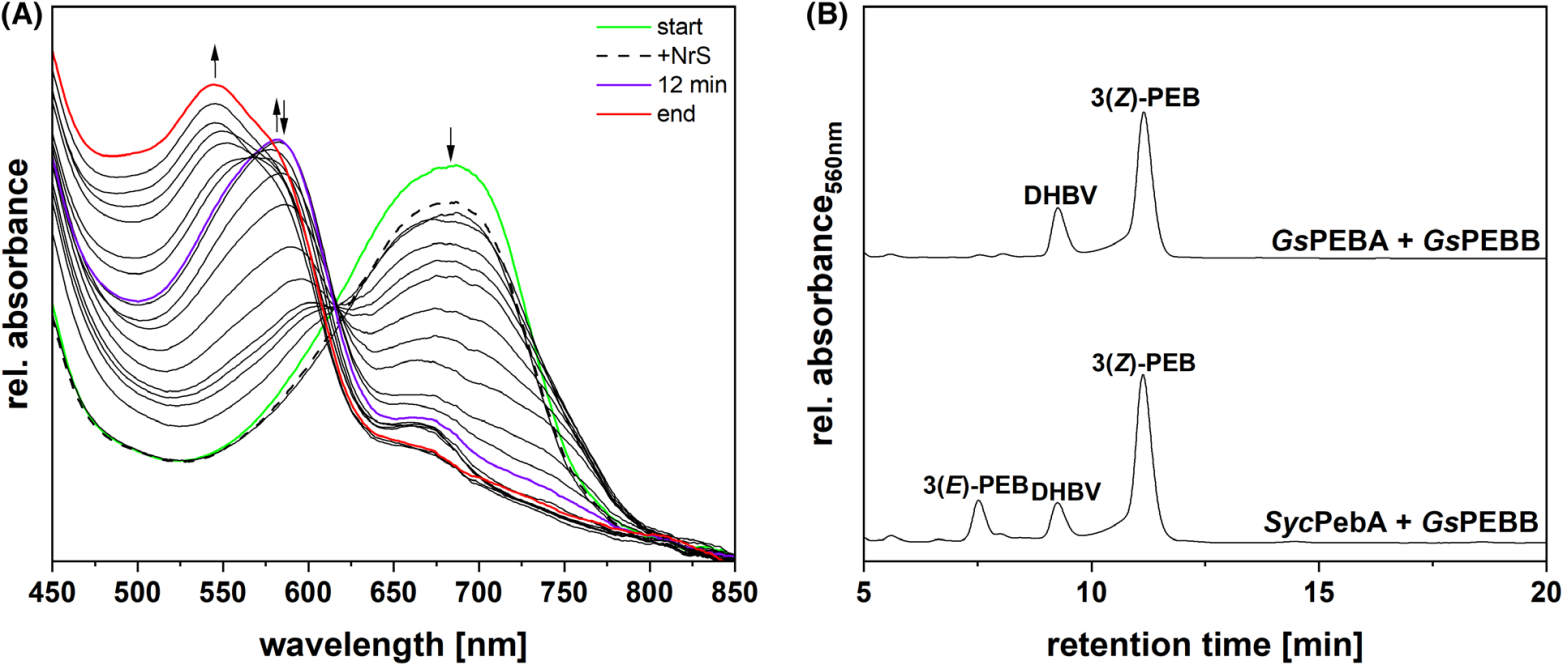
Coupled activity assay of recombinant *Gs*PEBA and *Gs*PEBB and identification of reaction products. (A) Spectra of an anaerobic bilin reductase activity assay using both recombinant *Gs*PEBA and *Gs*PEBB in a coupled approach, with BV as the substrate (*n* = 2). The total reaction time was 40 min, with spectra recorded at 30 s intervals. For ease of understanding, only the most relevant spectra are displayed. The arrows indicate the progression of absorbance during the reaction. The green spectrum corresponds to the ‘binding spectrum’, recorded upon incubation of BV with *Gs*PEBA and *Gs*PEBB. The dashed line represents the first spectrum recorded after initiating the reaction via the addition of the NrS. The spectra recorded during the reaction are shown as solid black lines; the violet spectrum represents the product formed after 12 min, while the final spectrum is colored in red. A 61‐point Savitzky–Golay filter was applied to smooth the curves. (B) HPLC analysis of the reaction products (*Gs*PEBA + *Gs*PEBB) (*n* = 1, as one analysis was enough to unequivocally identify the products). The products were separated on a reversed‐phase 5 μm C18 Luna column (Phenomenex), with a mobile phase consisting of 50% acetone (v/v) and 50% 20 mm formic acid (v/v), at a flow rate of 0.6 mL·min^−1^. Absorbance was monitored continuously at 560 nm. *Syc*PebA + *Gs*PEBB refers to the products of a coupled assay employing the PebA of *Synechococcus* sp. WH8020 and *Gs*PEBB, with BV as the substrate.

We considered two possibilities: first, that *Gs*PEBB might synthesize PCB from BV directly; and second, that the two FDBRs might produce PCB when acting together. The latter hypothesis is consistent with the presence of PEB:PCB isomerase activity in a *G. sulphuraria* enriched protein fraction with MW > 60 kDa, matching the size of a two‐FDBR complex [21]. We therefore examined the reaction(s) occurring when *Gs*PEBB and *Gs*PEBA were combined in a two‐enzyme activity assay (Fig. 6). Both enzymes were added to the reaction mix from the start, in equimolar amounts, and BV was used as substrate. The spectrum obtained following the addition of the enzymes and substrate displayed a peak at ~ 680 nm (Fig. 6A: start), which decreased in absorbance after the NrS was added (Fig. 6A: +NrS). After the initial formation of a product with an absorbance at ~ 580 nm (Fig. 6A: 12 min), the enzymes ultimately converted the substrate to a ~ 545 nm absorbing product (Fig. 6A: end). HPLC analysis of the final product revealed the presence of 15,16‐DHBV and PEB (Fig. 6B: *Gs*PEBA + *Gs*PEBB), mirroring the outcome of an equivalent reaction using PebA from *Synechococcus* sp. WH 8020 (*Syc*PebA) and *Gs*PEBB (Fig. 6B: *Syc*PebA + *Gs*PEBB). This result rules out these alternative hypotheses and reaffirms the need for an isomerase activity in *G. sulphuraria*.

**Fig. 7:**
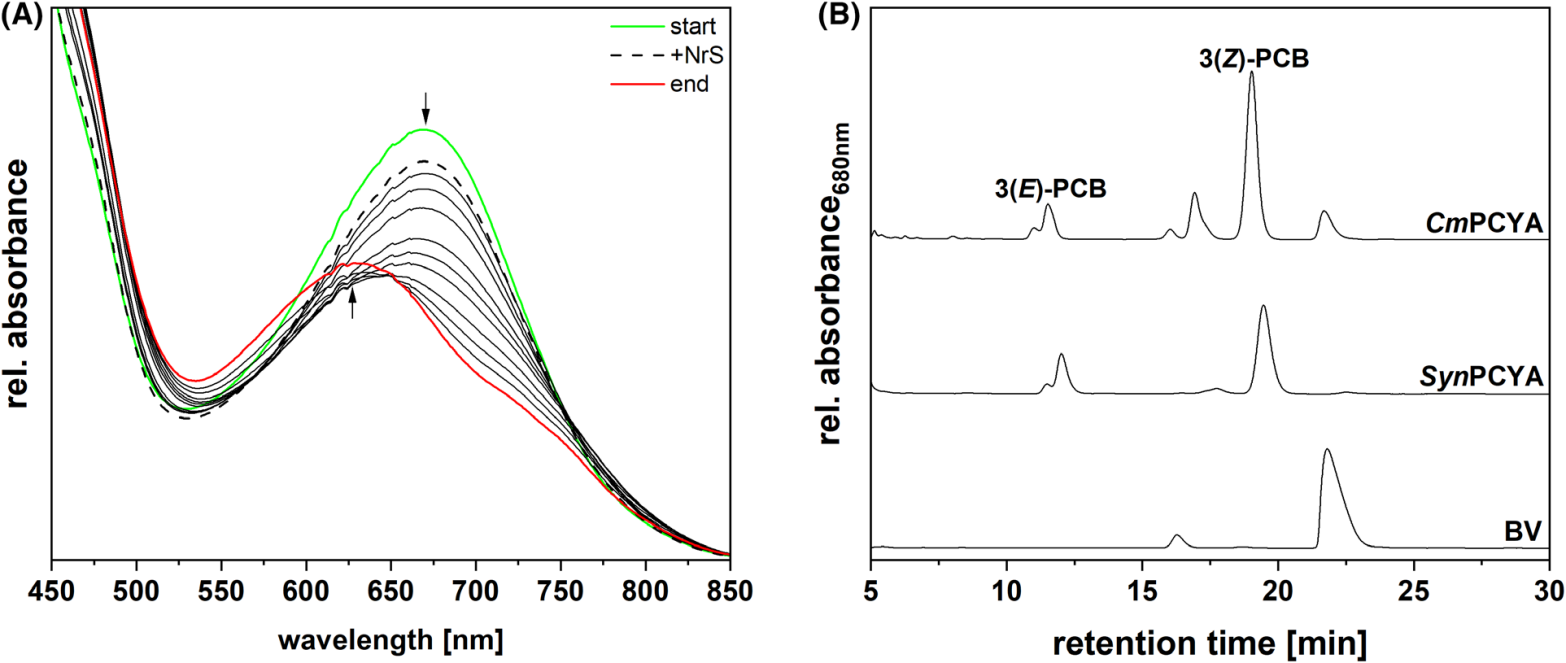
Investigation of the activity of recombinant *Cm*PCYA and identification of reaction products. (A) Spectra of an anaerobic bilin reductase activity assay using recombinant *Cm*PCYA and BV as the substrate (*n* = 2). The total reaction time was 10 min, with spectra recorded at 30 s intervals. For ease of understanding, only the most relevant spectra are displayed. The arrows indicate the progression of absorbance during the reaction. The green spectrum corresponds to the ‘binding spectrum’, recorded upon incubation of BV with *Cm*PCYA. The dashed line represents the first spectrum recorded after initiating the reaction via the addition of the NrS. The spectra recorded during the reaction are shown as solid black lines, while the final spectrum is colored in red. A 11‐points Savitzky–Golay filter was applied to smooth the curves. (B) HPLC analysis of the reaction products (*Cm*PCYA) (*n* = 1, as one analysis was enough to unequivocally identify the products). The products were separated on a reversed‐phase 5 μm C18 Luna column (Phenomenex), with a mobile phase consisting of 50% acetone (v/v) and 50% 20 mm formic acid (v/v), at a flow rate of 0.6 mL·min^−1^. Absorbance was monitored continuously at 680 nm. *Syn*PcyA refers to the product of an assay performed using *Synechocystis* sp. PCC 6803 PcyA and BV as the substrate; BV refers to the biliverdin standard.

We next examined PCB biosynthesis in *C. merolae*, a close relative of *G. sulphuraria* that possesses *PCYA* (see above). *Cm*PCYA would be expected to carry out direct, 4‐electron reduction of BV to PCB. Of several expression conditions, only the one in which the expression of pGEX‐6P‐1_*CmPCYA* was coupled with pGro7, to support protein folding, and the amount of the inducer and the incubation time were reduced proved to be successful in obtaining an active enzyme. Characterization of *Cm*PCYA confirmed that it is a *bona fide* PCYA. The reductase and the substrate formed a complex with an absorbance maximum at ~ 660 nm (Fig. 7A: start). Following the addition of the NrS, a modest decrease in absorbance of the complex peak could be observed (Fig. 7A: +NrS). Ultimately, *Cm*PCYA‐catalyzed BV reduction led to the formation of a product absorbing at ~ 620 nm (Fig. 7A: end). HPLC analysis confirmed *Cm*PCYA is consistent with its classification, catalyzing the reduction of BV to PCB (Fig. 7B: *Cm*PCYA). Furthermore, the pigment eluting at ~ 17 min potentially indicated the presence of 18^1^,18^2^‐DHBV as the intermediate, as has been observed for cyanobacterial PcyA [33, 72]. These results thus demonstrate that the rarely occurring rhodophyte PCYA sequences can indeed carry out direct PCB synthesis.

## Discussion

Although they are the basal clade of Rhodophyta, the Cyanidiophyceae exhibit a dark green coloration due to the absence of APC/PC in their PBS, suggesting that PCB is the sole bilin chromophore present. However, a study conducted over 30 years ago indicated that PCB biosynthesis may occur via PEB as an intermediate, via an isomerization reaction. Through fractionation of *G. sulphuraria* extracts, we confirmed the presence of the isomerase activity proposed in 1991. Specifically, a protein‐enriched fraction of *G. sulphuraria* with a molecular weight > 60 kDa was shown to catalyze this conversion in an enzyme‐dependent manner. Nevertheless, the presence of a putative PEB‐to‐PCB isomerase was proposed prior to the discovery of FDBRs, the enzymes now known to catalyze bilin biosynthesis. Since it could not be confirmed whether the observed activity was due to a novel, Rhodophyte‐specific isomerase or unexpected behavior of a known FDBR, a comprehensive analysis of FDBRs from well‐characterized, early branching Rhodophytes was undertaken. On the basis of the pigment composition, Cyanidiophyceae should encode only *PCYA*, responsible for synthesizing PCB to be subsequently bound to APC and PC, whereas other rhodophytes would also have *PEBA* and *PEBB* genes. This simple expectation does not hold true, providing the impetus for this study. Consistent with previous studies, our phylogenetic analysis demonstrates that rhodophyte PCYA sequences are only found in the early branching genera *Cyanidioschyzon* and *Cyanidiococcus*, along with a sequence in one of the two available genomes assigned to *Cyanidium*. All other rhodophyte genomes examined to date have only *PEBA* and *PEBB*, despite the need for PCB chromophores in the APC core and PC of rhodophyte PBS [73, 74]. Our phylogenetic analysis also provides several other insights. First, there is an apparent discrepancy in the two available genomes classified as *Cyanidium caldarium*: strain UTEX 2393, with genome accession GCA_019693505.1, has *PEBA* and *PEBB* and resembles *Galdieria* spp., but a single‐cell genome with accession GCA_026184775.1 instead has *PCYA*. It will thus be interesting to see the FDBR composition as other nuclear genomes become available for early diverging rhodophytes, such as the recently described Cavernulicolales [75]. We also observe that all three rhodophyte FDBRs are associated with candidate cryptophyte orthologs (Fig. 4), in contrast to the FDBRs found in other secondary algae with plastids derived from endosymbiosis with red algae [32, 42]. As in rhodophytes, cryptophytes have frequently lost PCYA. Cryptophyte PCYA proteins form a clade but are found in diverse genera that are not closely related to each other, consistent with acquisition of all three FDBRs at establishment of secondary endosymbiosis with subsequent loss. Our studies therefore indicate that the ancestral rhodophyte would also have had all three cyanobacterial FDBRs and that the loss of PCYA occurred early in rhodophyte evolution but after cryptophyte secondary endosymbiosis (Fig. S6). Hence, even the early branching *G. sulphuraria* has an apparent need for an isomerase to convert PEB into PCB for light harvesting.

**Fig. 8:**
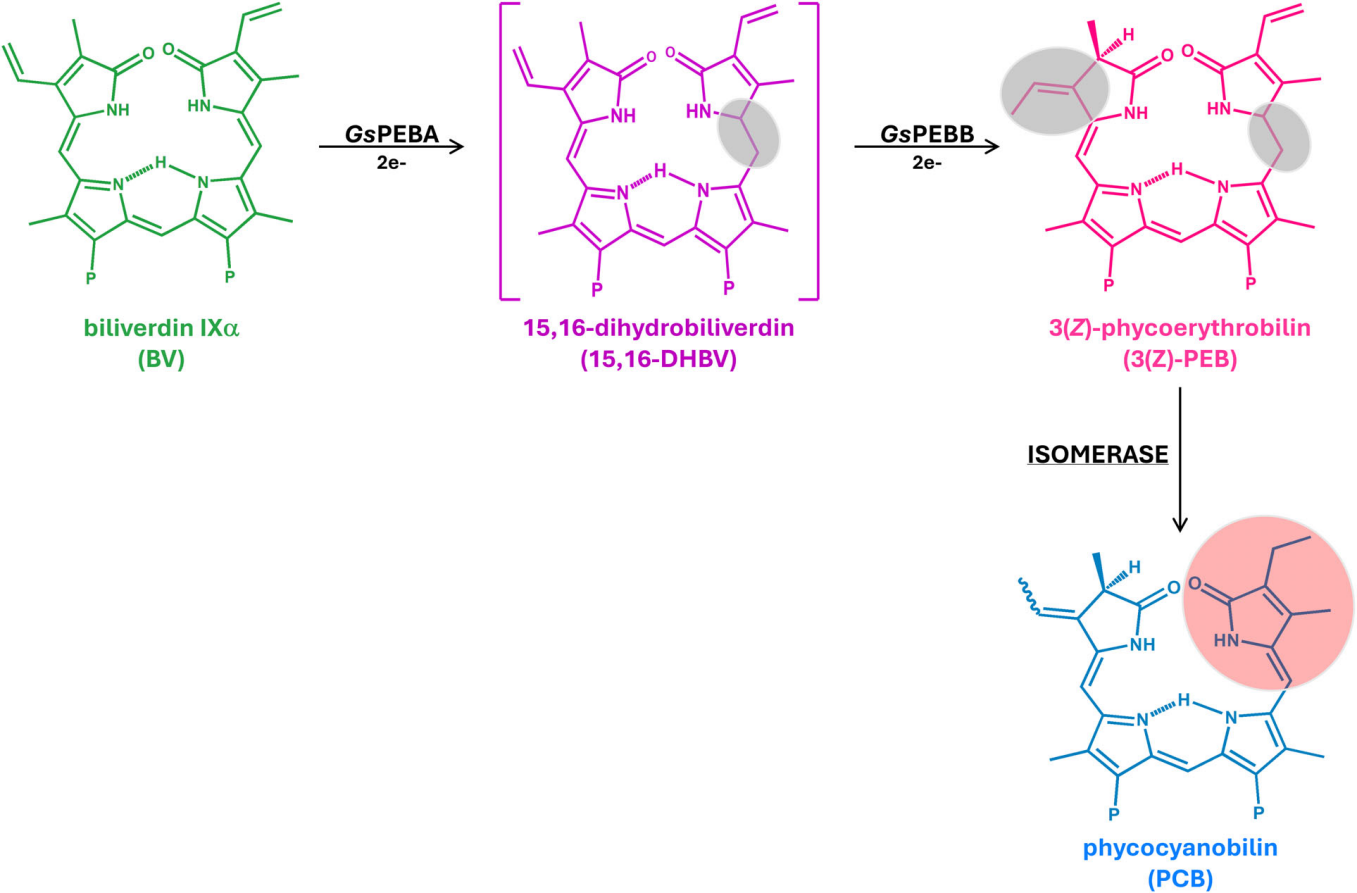
Proposed PCB biosynthesis pathway in *Galdieria sulphuraria*. *Gs*PEBA catalyzes the 2e^−^ reduction of BV to 15,16‐DHBV. *Gs*PEBB subsequently reduces 15,16‐DHBV to mostly 3(*Z*)‐PEB, with small traces of the 3(*E*) isomer. ‘P’ indicates the propionate side chains. BV reduction sites are highlighted in gray. 3(*Z*)‐PEB is the substrate for an isomerase acting on the C18^1^=C18^2^ double bond. Isomerization site is highlighted in red.

Although the two FDBRs, *Gs*PEBA and *Gs*PEBB, are classified phylogenetically according to their predicted activities, past studies have repeatedly shown that biochemical analyses are essential to confirm their final activities [31, 32, 37, 54]. We therefore confirmed the activity of representative PCYA, PEBA, and PEBB proteins from early branching rhodophytes. PEBA and PEBB from *G. sulphuraria* were proven both in individual and in coupled assays to be responsible for the production of only 15,16‐DHBV and PEB, respectively. Therefore, production of PCB would be expected to use PEB as a precursor because both bilins are at the same oxidation state and the reaction would thus be a simple isomerization reaction as proposed by Beale and Cornejo (Fig. 8) [21]. Consistent with this hypothesis, the kinetics of PEB loss and PCB appearance in the isomerase assay are indistinguishable. The same pathway is likely to be present in more complex Rhodophytes (Rhodophytina) as well. In such organisms, PE is also present in the PBS [73, 74], but PCB would still be required for energy transfer from PE to PC and then APC. The presence of PE explains the existence of *PEBA* and *PEBB* in these organisms, yet no *PCYA* is encoded for direct PCB biosynthesis starting from BV, again requiring an isomerase activity.

We also considered the possibility that PCB formation might proceed via the action of a bilin lyase‐isomerase. In this model, a PEB‐specific lyase‐isomerase carries out isomerization of PEB to PCB and attachment to PC and APC subunits in one step. Such mechanisms are typical of cyanobacteria, where the synthesis of PUB and phycoviolobilin (PVB) and their respective attachment to PE and phycoerythrocyanin are based on lyase‐isomerases [76, 77, 78, 79, 80, 81, 82, 83]. Although bilin lyases and lyase‐isomerases remain poorly characterized in rhodophytes, blast searches led to the identification of the PC‐related enzymes CpcE/F, CpcS, and CpcT in both *C. merolae* and *G. sulphuraria* (Table S2). *G. sulphuraria* uniquely possesses CpcU, but this protein is thought to only function as a heterodimer with CpcS [84, 85, 86]. Hence, the apparent palette of bilin lyases is quite similar in both organisms, even though only one has a need for isomerase activity.

Future efforts to identify the bilin isomerase in *G. sulphuraria* will require further classical purification of the enzyme, followed by mass spectrometry‐based analysis and subsequent gene identification. While mass spectrometry may play a central role, the process could also benefit from the application of novel methodological approaches. These steps will be the focus of future work by our group.

## Materials and methods

### Chemicals

All chemicals used in this study were ACS grade or better. Biliverdin IXα was purchased from Frontier Scientific (Newark, DE, USA). Ferredoxin (PetF) and Ferredoxin‐NADP^+^ reductase (FNR or PetH) were recombinantly expressed and purified as described elsewhere [35, 36]. HPLC‐grade acetone, formic acid, and acetonitrile were purchased from VWR Chemicals (Darmstadt, Germany). Blue Sepharose™ 6 Fast Flow and Superdex™ 75 10/300 GL were purchased from GE Healthcare (Düsseldorf, Germany). Other reagents were purchased from Sigma‐Aldrich (Taufkirchen, Germany).

### Plasmids and primers

Gene fragments (accession codes in Table 1) were purchased from Twist Bioscience (South San Francisco, CA, USA) and Integrated DNA Technologies, Inc. (Coralville, IA, USA). The plasmids in Table 1 were generated via Gibson assembly [87], employing primers listed in Table 2, except for pASK‐IBA45(+)_*GsPEBB*. In that case, the gene was subcloned from the expression vector pCR2.1 (Eurofins Genomics Germany GmbH, Ebersberg, Germany) with *Eco*RI and *Not*I restriction sites at the 5′ and 3′ end, respectively. *GsPEBB* was codon‐optimized for *Escherichia coli* using the algorithm provided by the Eurofins Genomics website, whereas *GsPEBA* was codon‐harmonized using the charming algorithm (http://www.codons.org/codons.html) [88].

**Table 1:** Plasmids.

Plasmid	Features	Accession code
pET28a_*GsPEBA*	pET‐28a(+) derivative carrying the codon‐harmonized *PEBA* gene sequence from *Galdieria sulphuraria* (from L70 to S302); N‐terminal His_6_‐tag	Gasu_55890 (EnsemblPlants)
pASK‐IBA45(+)_*GsPEBB*	pASK‐IBA45(+) derivative carrying the codon‐optimized *PEBB* gene sequence from *G. sulphuraria* (from N64 to T306); N‐terminal Strep‐tag II	Gasu_58250 (Ensembl Plants)
pGEX‐6P‐1_*CmPCYA*	pGEX‐6P‐1 derivative carrying the original *PCYA* gene sequence from *Cyanidioschyzon merolae*; N‐terminal GST‐tag	CMG110C (Ensembl Plants)

**Table 2:** Primers.

Oligonucleotide	Sequence (5′ → 3′)
pET28a_*GsPEBA*_fwd	CTGGTGCCGCGCGGCAGCCAGGATTCGAACAGCTTAGTTAG
pET28a_*GsPEBA*_rev	GTCGACGGAGCTCGAATTCGTTAGGTCTTGTTATCAAGACT
pASK‐IBA45(+)_*GsPEBB*_fwd	CGAATTCGTTGTATTCCCCGTTC
pASK‐IBA45(+)_*GsPEBB*_rev	GGGAAGCTTTTAGGTGCTCAGTTTG
pGEX‐6P‐1_*CmPCYA*_fwd	CCCGGAATTCCCGGGTCGACAAATGCGCTTGCGCGG
pGEX‐6P‐1_*CmPCYA*_rev	GTCAGTCACGATGCGGCCGCCTAGTTCGCTGCCAGCAC

### *Galdieria sulphuraria* cultivation and cell disruption

Growth medium (composition listed in Table S3) was inoculated with a single colony of *G. sulphuraria* 074 W and incubated under continuous light (50 μE m^−2^ s^−1^) at 37 °C and 120 r.p.m. (Innova^®^ 44R; New Brunswick Scientific, Edison, NJ, USA). After reaching the exponential phase, cells were harvested by centrifugation at 3100 ***g*** for 10 min (Eppendorf 5810 R, Rotor A‐4‐62; Hamburg, Germany) and stored at −20 °C.

Approximately 7 g cells were thawed and washed three times each with H_2_O and then with Extraction buffer (50 mm HEPES; 5 mm EDTA; 10% (v/v) Glycerol; pH 7.3). Cells were resuspended in 40 mL Extraction buffer and lysed via two passages through a French^®^ Pressure Cell Press (FA‐078; Thermo Fisher, Waltham, MA; USA) at 10 000 PSI. The lysate was centrifuged (50 000 ***g***, 30 min, 4 °C) to remove cell debris, and the supernatant was collected for further processing.

### *Galdieria sulphuraria* protein fraction enrichment

(NH_4_)_2_SO_4_ was finely grounded using a mortar and pestle and then added to the lysate to achieve a 30% saturating concentration. The solution was stirred at 4 °C for 1 h and subsequently centrifuged (50 000 ***g***, 1 h, 4 °C, Thermo Scientific ^TM^ Sorvall LYNX 6000, Rotor T29). The pellet was discarded, and (NH_4_)_2_SO_4_ was added to a saturating concentration of 45%. The solution was again stirred at 4 °C for 1 h and centrifuged (50 000 ***g***, 30 min, 4 °C). The pellet was dissolved in 20 mL cold Assay buffer (25 mm HEPES; 1 mm MgCl_2_; 10% glycerol (v/v); pH 7.3) and dialyzed at 4 °C overnight against Assay buffer to remove (NH_4_)_2_SO_4_. Affinity chromatography was performed using an ÄKTA pure™ chromatography system (GE Healthcare) equipped with a Blue Sepharose™ 6 Fast Flow column (GE Healthcare) pre‐equilibrated with Assay buffer. The dialyzed protein solution obtained after the last ammonium sulfate precipitation was filtered using a Phenex™‐PTFE 0.45 μm syringe filter (Phenomenex, Torrance, CA, USA) and manually loaded into the ÄKTA system with a Superloop™ (GE Healthcare). Chromatography was performed at 4 °C at a flow rate of 1 mL·min^−1^. Protein elution was achieved using Elution buffer (25 mm HEPES; 1 mm MgCl_2_; 10% glycerol (v/v); 1 m NaCl; pH 7.3), and 5 mL fractions were collected. (NH_4_)_2_SO_4_ was added to the appropriate elution fraction at a saturating concentration of 70%, and the solution was stirred at 4 °C for 1 h and then subsequently centrifuged (50 000 ***g***, 30 min, 4 °C). The precipitated proteins were dissolved in 5 mL cold Assay buffer to a concentration of 2.5 mg·mL^−1^ for size exclusion chromatography (SEC). Chromatography was performed on the ÄKTA™ pure 25 system equipped with a Superdex™ 75 10/300 GL column (GE Healthcare) pre‐equilibrated with Assay buffer. SEC was performed at 4 °C at a flow rate of 1 mL·min^−1^, and 2 mL fractions were collected. Fractions containing the desired size range of proteins were pooled and concentrated using Amicon® Ultra 4 mL Centrifugal Filters with a MWCO of 10 kDa (Merck KGaA, Darmstadt, Germany).

### Isomerase activity assay

To assess PEB:PCB isomerase activity, the purified and concentrated protein fraction was incubated with 14 μm PEB, obtained via methanolytic cleavage from *Porphyridium purpureum* PE, for 80 min at 30 °C. Isomerase activity was monitored via UV–Vis spectroscopy using an Agilent 8453 spectrophotometer (Santa Clara, CA, USA). The initial spectrum was recorded in the absence of PEB; the following one immediately after the addition of PEB, and subsequent spectra were recorded every 10 min thereafter. Negative controls were performed either via the substitution of the enriched fraction with assay buffer or heat inactivation. In the latter, the enriched fraction was heated to 95 °C for 10 min and subsequently centrifuged at 10 000 ***g***. The denatured, precipitated protein pellet was discarded, while the supernatant was used to perform the activity assay.

Following the activity assay, the reaction products were prepared for HPLC as described for the anaerobic bilin reductase assay products.

### Bioinformatics and phylogenetic analysis

To identify additional candidate FDBR sequences, we conducted blast [89] searches against the NCBI and DOE‐IMG databases. We also conducted blast searches against additional metagenomic resources [62, 63, 65, 90] that were downloaded and used to construct local blast databases. We then constructed a multiple sequence alignment in mafft v7.450 [91] using the E‐INS‐i algorithm (equivalent to the command‐line settings ‐‐genafpair ‐‐maxiterate 16 ‐‐clustalout ‐‐reorder). Alignment positions having gaps at ≥ 5% of the total sequences were removed using an in‐house script [32] to yield a final alignment having 337 sequences and 168 characters. All sequences were ≥ 90% complete after gap removal. The final alignment was then used to infer phylogenies in phyml‐3.3 [66] and iq‐tree 3.0.1 (https://iqtree.github.io/). Tree inference in phyml used the WAG substitution model and the SPR search algorithm with 100 bootstraps and calculation of the TBE [67] as implemented in phyml (command‐line settings ‐m WAG ‐d aa ‐s SPR ‐a e ‐c 4 ‐v e ‐o tlr ‐b 100 ‐‐tbe). The parallel version of phyml was used with four cores running under OpenMPI installed via HomeBrew (https://brew.sh/). Tree inference in iq‐tree used automatic model selection with modelfinder [68] (command‐line settings: ‐T AUTO ‐msub nuclear), and supports were evaluated using ufboot and sh‐alrt [66, 69]. Trees were visualized and processed in treeviewer [70].

Searches for *G. sulphuraria* FDBRs used a broad panel of known or candidate FDBRs as queries (Table S1), including sequences detected during the above process, along with Arabidopsis RCCR and a candidate bacterial CPO. Searches used blastp 2.17.0+ [89] and hmmer 3.4 (phmmer searches using a single query; hmmer.org) using default settings. For blast searches, an *e*‐value of 1e‐5 was taken as the threshold of significance, whereas the default internal threshold was taken for hmmer.

### Production and purification of recombinant proteins

For production of recombinant His‐tagged *Gs*PEBA, 3 L of LB medium supplemented with 50 μg·mL^−1^ kanamycin was inoculated 1:100 with an overnight culture of *E. coli* BL21(DE3) carrying pET28a_*GsPEBA*. The cultures were grown at 37 °C and 100 r.p.m. (New Brunswick™ INNOVA^®^ 44) to an OD_600_ of 0.4–0.6. The temperature was decreased to 17 °C and gene expression was induced by supplementing with 1 mm isopropyl‐β‐thiogalactoside (IPTG). The cultures were incubated under shaking for 19 additional hours and harvested by centrifugation for 10 min at 17 000 ***g*** and 4 °C (Sorvall LYNX 6000, Rotor F9). The pellet containing His‐tagged *Gs*PEBA was resuspended in His‐Binding buffer (20 mm sodium phosphate pH 7.4; 500 mm NaCl) at a ratio of 3 mL buffer·g^−1^ wet cell weight. After the addition of 5 μg·mL^−1^ DNaseI (AppliChem GmbH, Darmstadt, Germany) and 1 mg·mL^−1^ lysozyme (Sigma‐Aldrich), the suspension was kept on ice for 30 min. The cells were disrupted using a microfluidizer (LM 10 Microfluidizer, Microfluidics™; 3 cycles at 15 000 psi) and centrifuged for 45 min at 50 000 ***g*** and 4 °C (Sorvall LYNX 6000, Rotor T29). The crude extract was loaded onto a gravity flow column containing 2 mL of TALON^®^ Superflow™ resin (Cytiva, Freiburg im Breisgau, Germany). After washing with 10 column volumes (CV) of His‐binding buffer, elution was performed using 4 CV of His‐elution buffer (20 mm sodium phosphate pH 7.4; 500 mm NaCl; 500 mm imidazole).

For production of recombinant Strep‐tagged *Gs*PEBB, 3 L LB medium supplemented with 100 μg·mL^−1^ ampicillin was inoculated 1:100 with an overnight culture of *E. coli* BL21(DE3) carrying pASK‐IBA45(+)_*GsPEBB*. Cells were grown at 37 °C and 100 r.p.m. (New Brunswick™ INNOVA^®^ 44) to an OD_600_ of 0.4–0.6. The temperature was decreased to 17 °C and gene expression was induced supplementing 200 ng·mL^−1^ anhydrotetracycline (AHT). The cultures were incubated under shaking for 19 additional hours and harvested by centrifugation for 10 min at 17 000 ***g*** and 4 °C (Sorvall LYNX 6000, Rotor F9). The pellet containing Strep‐*Gs*PEBB was resuspended in Strep‐Binding buffer (100 mm Tris/HCl pH 8; 300 mm NaCl; 1 mm EDTA) at a ratio of 3 mL buffer·g^−1^ wet cell weight. After the addition of 5 μg·mL^−1^ DNaseI and 1 mg·mL^−1^ lysozyme, the suspension was kept on ice for 30 min. The cells were disrupted, centrifuged using the same conditions as for *Gs*PEBA (see above). The crude extract containing Strep‐*Gs*PEBB was loaded onto a gravity flow column containing 3 mL of Strep‐Tactin^®^ Sepharose^®^ (IBA Lifesciences GmbH, Göttingen, Germany). After washing with 10 CV of Strep‐Binding buffer, elution was performed using 4 CV of Strep‐Elution buffer (100 mm Tris/HCl pH 8; 300 mm NaCl; 1 mm EDTA; 2.5 mm Desthiobiotin).

For the production of recombinant GST‐tagged *Cm*PCYA, 2 L of LB medium supplemented with 100 μg·mL^−1^ ampicillin and 34 μg·mL^−1^ chloramphenicol was inoculated 1 : 100 with an overnight culture of *E. coli* BL21(DE3) carrying pGEX‐6P1_*CmPCYA* and pGro7, to support protein folding. Cells were grown at 37 °C and 100 r.p.m. (New Brunswick™ INNOVA^®^ 44) to an OD_600_ of 0.4–0.6. The temperature was decreased to 17 °C and gene expression was induced by the addition of 0.05 mm IPTG. The cultures were incubated under shaking for three additional hours and harvested by centrifugation for 10 min at 17 000 ***g*** and 4 °C (Sorvall LYNX 6000, Rotor F9). The pellet containing GST‐tagged *Cm*PCYA was resuspended in phosphate‐buffered saline (PBS: 140 mm NaCl; 10 mm Na_2_HPO_4_; 2.7 mm KCl; 1.8 mm KH_2_PO_4_; pH 7.4) supplemented with 0.05% Triton X‐100 at a ratio of 3 mL buffer·g^−1^ wet cell weight. After the addition of 5 μg·mL^−1^ DNaseI and 1 mg·mL^−1^ lysozyme, the suspension was kept on ice for 30 min. The cells were disrupted by sonication (Bandelin Sonopuls HD 2200; tip KE76; 5′, 5″ pulses, 10″ pauses; cycle 5/10; ≈ 50% power output) and centrifuged for 45 min at 4 °C and 50 000 ***g*** (Sorvall™ LYNX™ 6000, rotor T29‐8). The crude extract containing GST‐*Cm*PCYA was loaded onto a gravity flow column containing 2 mL of Protino^®^ Glutathione Agarose 4B (Macherey‐Nagel GmbH & Co. KG, Düren, Germany). After washing with 10 CV of PBS, elution was performed using 3 CV of GST‐Elution buffer (50 mm Tris/HCl pH 8; 10 mm reduced Glutathione).

After elution, fractions for each protein were pooled and dialyzed overnight against TES‐KCl buffer (25 mm TES/KOH pH 7.5; 100 mm KCl; 10%_v/v_ glycerol).

### Anaerobic bilin reductase assay

The anaerobic bilin reductase activity assays were conducted following the method outlined by Ledermann and colleagues with slight modifications [56]. Recombinant ferredoxin PetF from the cyanophage P‐SSM2 served as the electron donor at a final concentration of 1 μm. PetF was reduced using recombinant FNR from *Synechococcus* sp. PCC 7002 at a final concentration of 0.01 μm [35]. The reaction was initiated by adding an NADPH‐regenerating system consisting of 65 mm glucose‐6‐phosphate, 8.2 μm NADP^+^ and 11 U·mL^−1^ glucose‐6‐phosphate dehydrogenase. Absorption spectra were measured using an Agilent 8453 spectrophotometer at a constant temperature of 20 °C. The reaction was stopped by 10‐fold dilution of the reaction mix into ice cold 0.1% trifluoroacetic acid (TFA; v/v). Products were then isolated via solid phase extraction using Sep‐Pak^®^ C18 Plus Light cartridges (Waters, Milford, MA, USA) and freeze‐dried using an Alpha 2–4 LSC plus lyophilizer (Martin Christ GmbH, Osterode, Germany) prior to HPLC analysis.

### HPLC analyses

Isolated assay products were analyzed using an Agilent 1100 series HPLC system equipped with a Luna^®^ 5 μm reversed‐phase C18‐column (Phenomenex) and a diode‐array detector. The lyophilized samples were dissolved in 10 μL DMSO prior to analysis. The mobile phase consisted of 50% (v/v) acetone and 50% (v/v) aqueous 20 mm formic acid, flowing at 0.6 mL·min^−1^. Reaction products were identified by comparing their retention times with known standards and through full‐spectrum analysis of the elution peaks.

## Conflict of interest

The authors declare no conflict of interest.

## Author contributions

FF, NCR, and NF‐D designed research; FF, JH, JMM, and NCR performed research; FF, NCR, and NF‐D wrote the paper. All authors read and approved the final version of the manuscript.

## Supporting information


**Fig. S1.** Overview of bilin reductases.
**Fig. S2.** Size exclusion chromatography of bovine serum albumin (BSA).
**Fig. S3.** Comparison of maximum‐likelihood phylogenetic analyses of FDBRs.
**Fig. S4.** Annotated phylogenetic analysis of FDBRs.
**Fig. S5.** Investigation of potential activity of recombinant *Gs*PEBB incubated with BV as substrate.
**Fig. S6.** Acquisition and loss of FDBRs in rhodophytes and cryptophytes.
**Table S1.** Identification of FDBR homologs in *Galdieria sulphuraria*.
**Table S2.** Comparison of phycobiliprotein lyases in *Galdieria sulphuraria* and *Cyanidioschyzon merolae*.
**Table S3.** *Galdieria sulphuraria* growth media and Trace elements composition.

## Data Availability

Raw data are available on DataDryad: https://doi.org/10.5061/dryad.hhmgqnksx.
