# Supplementary material for: Phycocyanobilin biosynthesis in Galdieria sulphuraria requires isomerization of phycoerythrobilin synthesized by bilin reductases
Source: FEBS J. 2026 Jan 10;293(10):2908–27. doi: 10.1111/febs.70391 (PMC13193486; doi:10.1111/febs.70391)
Supplement: Supplementary file 1 — Fig. S1. Overview of bilin reductases. Fig. S2. Size exclusion chromatography of bovine serum albumin (BSA). Fig. S3. Comparison of maximum‐likelihood phylogenetic analyses of FDBRs. Fig. S4. Annotated phylogenetic analysis of FDBRs. Fig. S5. Investigation of potential activity of recombinant GsPEBB incubated with BV as substrate. Fig. S6. Acquisition and loss of FDBRs in rhodophytes and cryptophytes. Table S1. Identification of FDBR homologs in Galdieria sulphuraria. Table S2. Comparison of phycobiliprotein lyases in Galdieria sulphuraria and Cyanidioschyzon merolae. Table S3. Galdieria sulphuraria growth media and Trace elements composition. [file FEBS-293-2908-s001.pdf]

Supporting Data

Phycocyanobilin biosynthesis in *Galdieria sulphuraria* requires isomerization of phycoerythrobilin synthesized by ferredoxin-dependent bilin reductases

Federica Frascogna[1,§], Nathan C. Rockwell[2,§] Jana Hartmann[1], Julie M. Mudler[1], Nicole Frankenberg-Dinkel[1*]

[1]*Department of Microbiology, RPTU Kaiserslautern-Landau, 67663 Kaiserslautern, Germany*

[2]*Department of Molecular and Cell Biology, One Shields Avenue, University of California at Davis, Davis, CA 95616 USA*

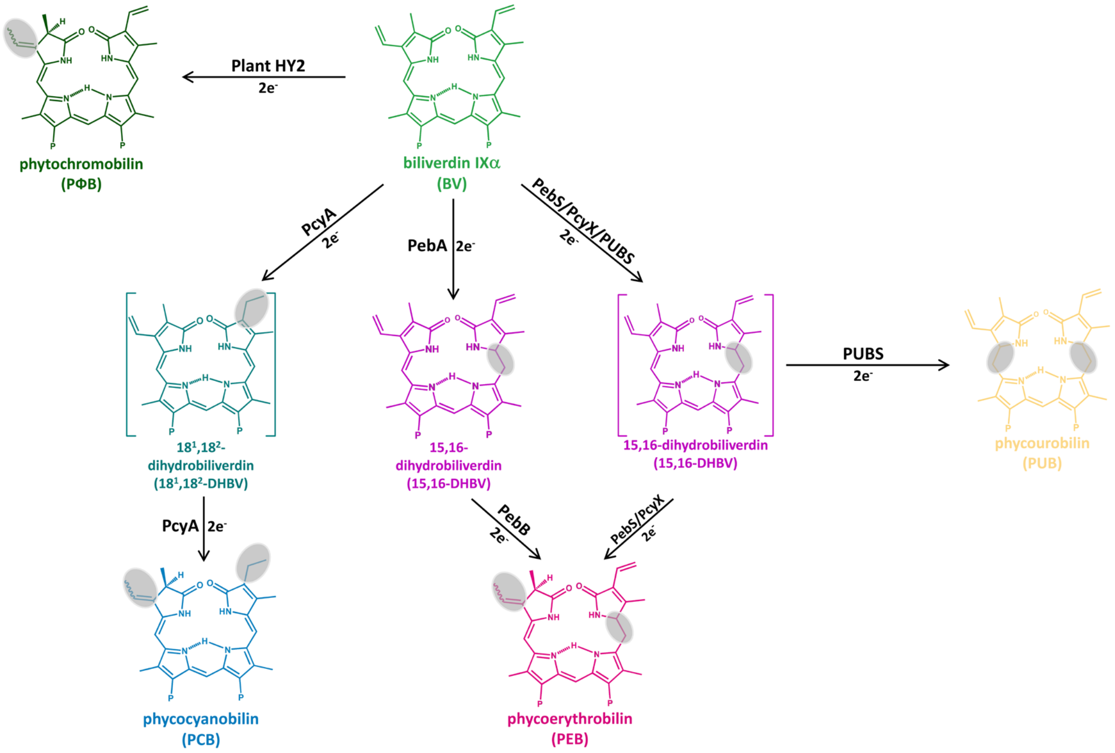

Supporting Figure S1. Overview of bilin reductases. Reduction sites are highlighted in grey. The plant enzyme phytochromobilin synthase (HY2) is responsible for the reduction of biliverdin (BV) A-ring to yield phytochromobilin (PΦB) [24,30]. A second type of HY2, the one of streptophyte algae, catalyzes the reduction of BV to phycocyanobilin (PCB), either via PΦB or 18^1,18^2-dihydrobiliverdin (18^1,18^2-DHBV) [31,32]. PcyA catalyzes the $4e^-$ reduction of BV first to the intermediate 18^1,18^2-DHBV and ultimately to PCB [33,34]. PebA reduces BV to 15,16-dihydrobiliverdin (15,16-DHBV), while PebB converts the latter to phycoerythrobilin (PEB) [35]. PebS and PcyX combines the activity of PebA and PebB in one enzyme [36,37]. PUBS, only found in Viridiplantae, catalyzes the $4e^-$ reduction of BV to phycourobilin, via 15,16-DHBV as intermediate [38,39].

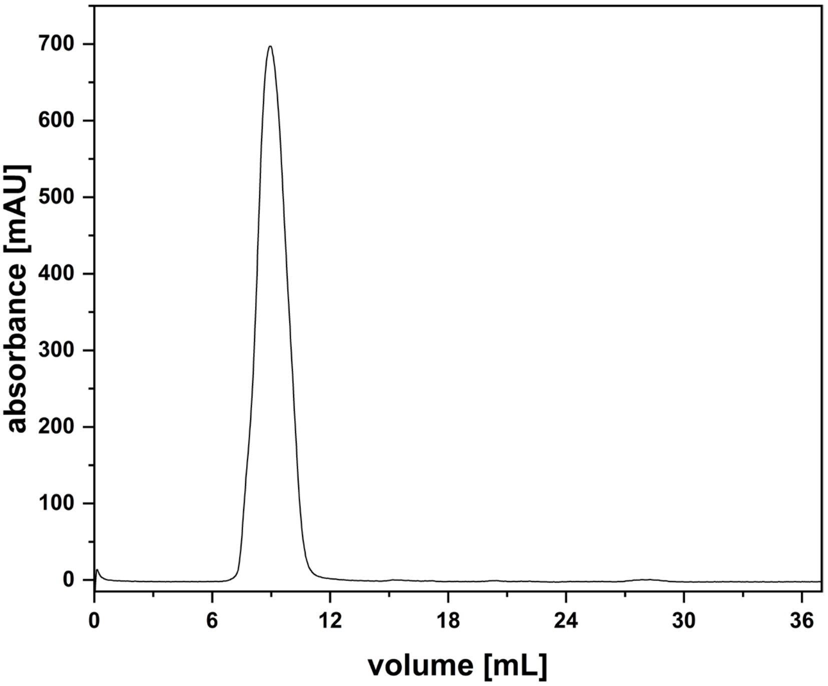

Supporting Figure S2. Size exclusion chromatography of bovine serum albumin (BSA).
Size exclusion chromatography was performed using the ÄKTA™ pure 25 purification system equipped with a Superdex™ 75 10/300 GL, equilibrated with "Assay buffer". Absorbance was continuously measured at 280 nm.

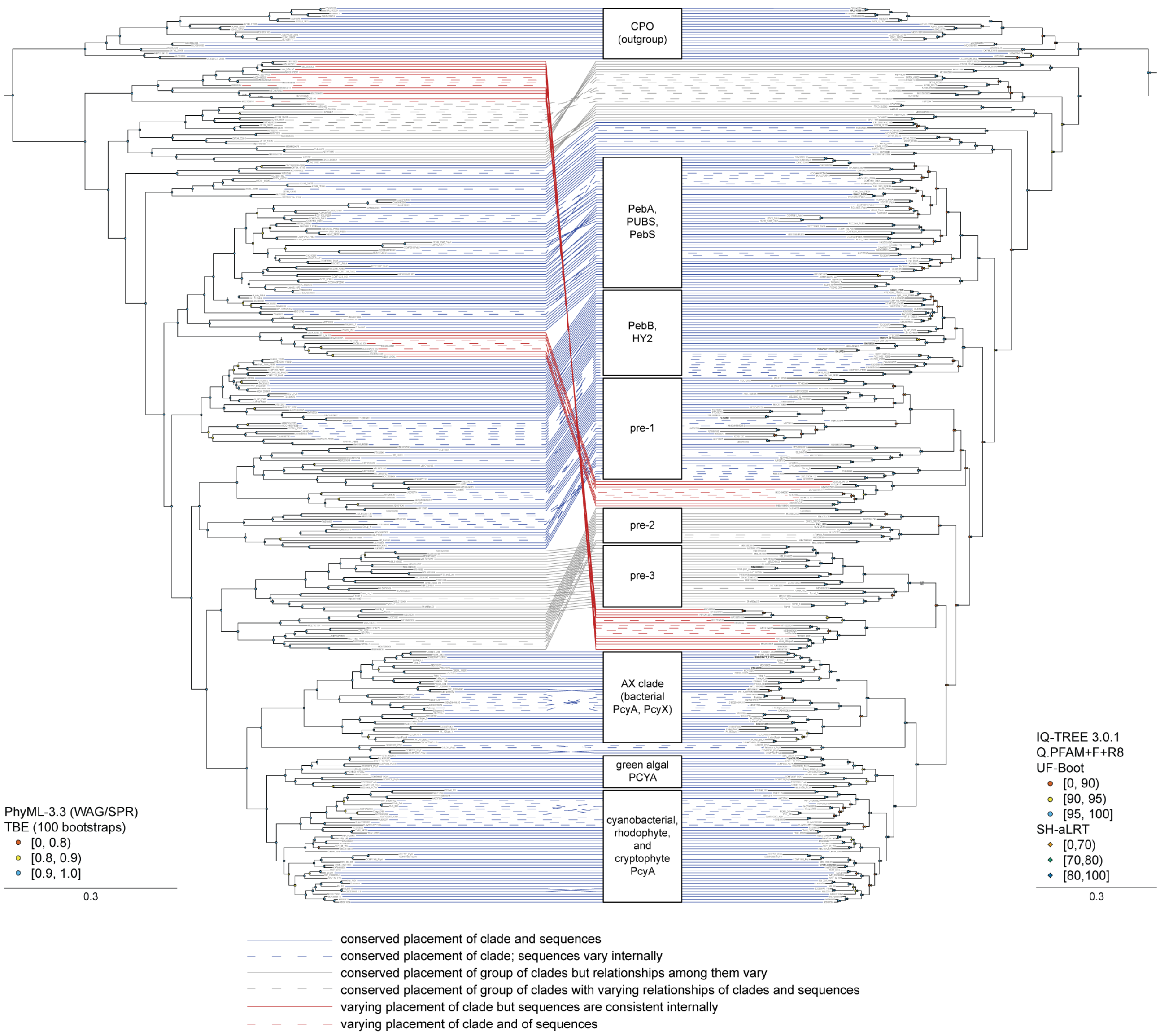

Supporting Figure S3. Comparison of maximum-likelihood phylogenetic analyses of FDBRs. Phylogenetic trees were inferred using either PhyML version 3.3.20250429 (left) or IQ-Tree version 3.0.1 (right) as described in the Methods. The two trees are compared in a tanglegram representation. Two clades were placed in significantly different places in the two analyses (dark red lines), and some other clades were placed in the same approximate branching order but with differences in their relationship to each other (grey lines). Other clades were found in the same branching order (blue lines). Sequences assigned to the same overall clade but having varying relationships within that clade are indicated using dashed lines. Supports are indicated symbolically; explicit SH-aLRT supports are in italics, and UFBoot supports are in bold italics. The original alignment, gap-trimmed input file, and output files from both software packages are available via DataDryad (DataDryad: https://doi.org/10.5061/dryad.hhmgqnksx).

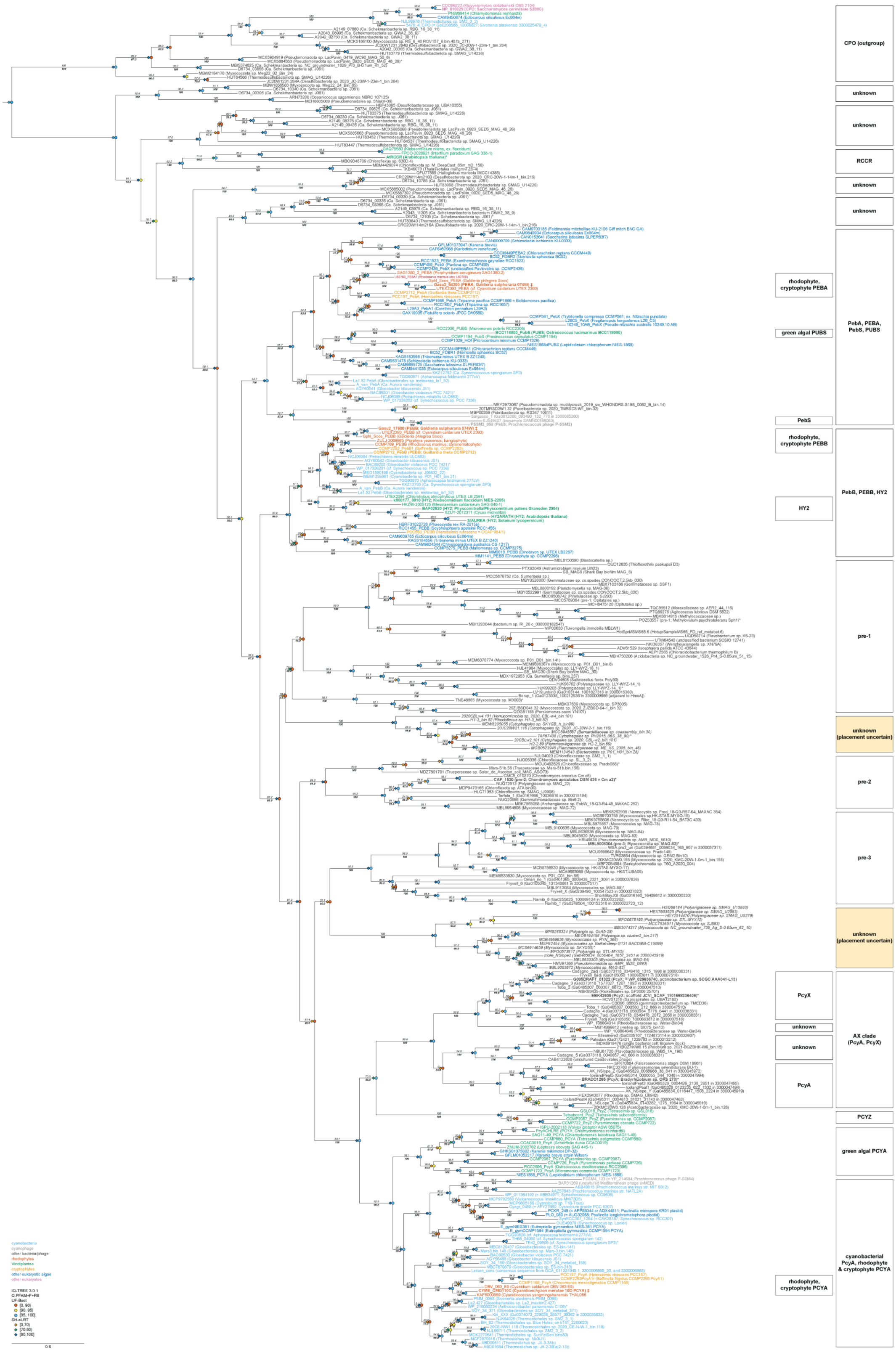

Supporting Figure S4. Annotated phylogenetic analysis of FDBRs. The phylogenetic tree inferred in IQ-Tree version 3.0.1 is shown with sequences colored by taxon as indicated and with accession information added. Clades are indicated, and characterized sequences are indicated in bold. Clades which were placed in significantly different locations in the PhyML tree (Supporting Figure S3) are indicated as “placement uncertain,” with sequences in italics. *, Sequences used in searching for cryptic FDBRs in *G. sulphuraria*; ‡, sequences characterized biochemically in the

current work. The original alignment, gap-trimmed input file, and output files are available via DataDryad (DataDryad: https://doi.org/10.5061/dryad.hhmgqnksx).

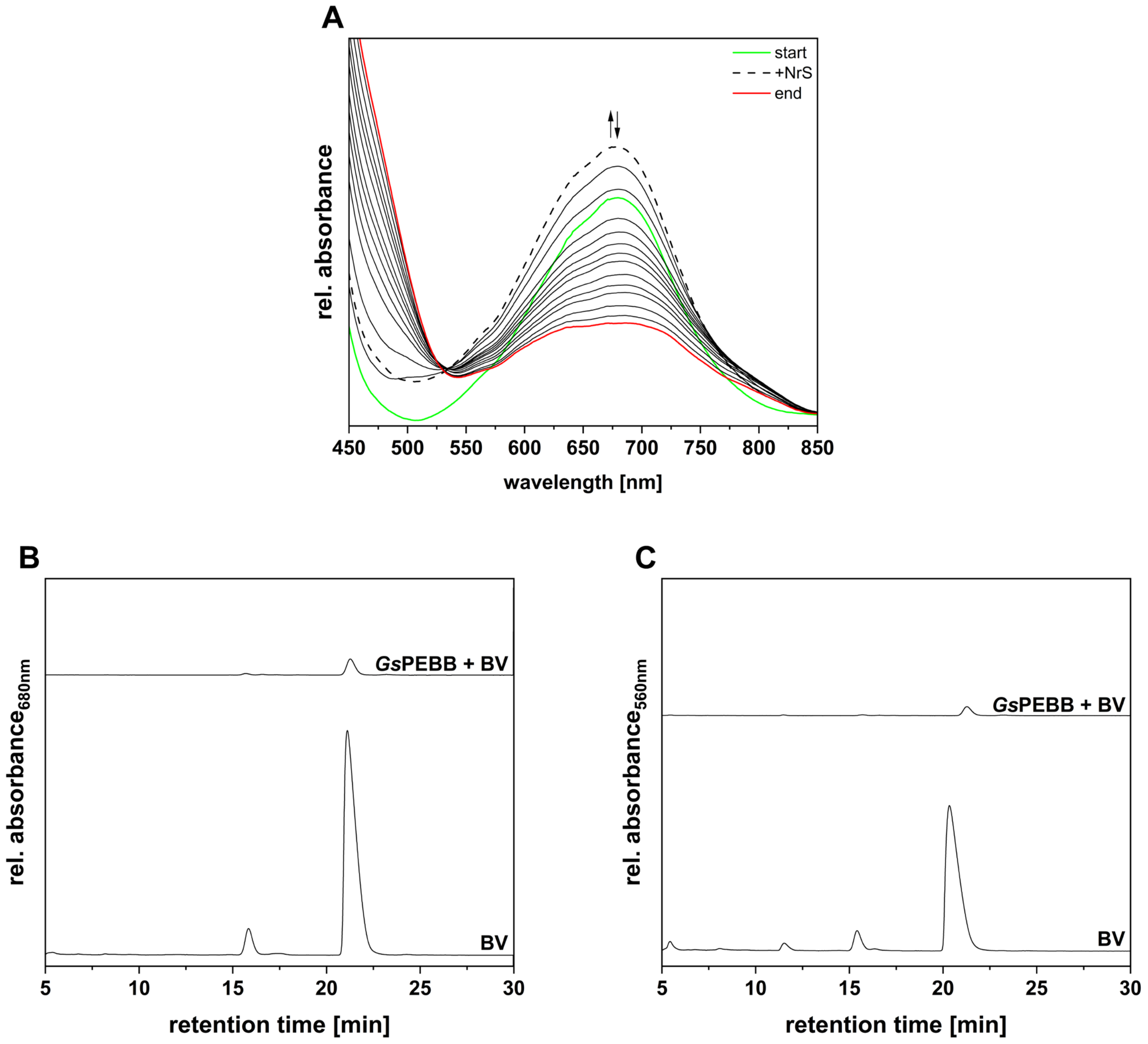

Supporting Figure S5. Investigation of potential activity of recombinant *Gs*PEBB incubated with BV as substrate.

(A) Spectra of an anaerobic bilin reductase activity assay using recombinant *Gs*PEBB and BV as the substrate. The total reaction time was 20 minutes, with spectra recorded at 30 s intervals. For ease of understanding, only the most relevant spectra are displayed. The arrows indicate the progression of absorbance during the reaction. The green spectrum corresponds to the “binding spectrum”, recorded upon incubation of BV with *Gs*PEBB. The dashed line represents the first spectrum recorded after initiating the reaction via the addition of the NrS (NADPH-regenerating system). The spectra recorded during the reaction are shown as solid black lines, while the final spectrum is colored in red. A 30-points Savitzky-Golay filter was applied to smooth the curves.

(B) HPLC analysis of the reaction products (*Gs*PEBB + BV). The products were separated on a reversed-phase 5 µm C18 Luna column (Phenomenex), with a mobile phase consisting of 50% acetone (v/v) and 50% 20 mM formic acid (v/v), at a flow rate of 0.6 mL/min. Absorbance was monitored continuously at 680 nm.

(C) HPLC analysis of the reaction products (*Gs*PEBB + BV). The products were separated on a reversed-phase 5 µm C18 Luna column (Phenomenex), with a mobile phase consisting of 50% acetone (v/v) and 50% 20 mM formic acid (v/v), at a flow rate of 0.6 mL/min. Absorbance was monitored continuously at 560 nm.

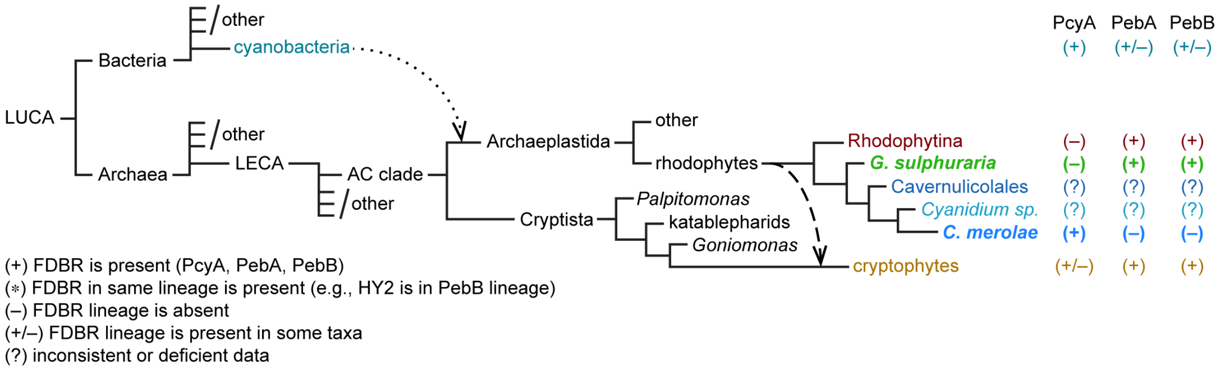

Supporting Figure S6. Acquisition and loss of FDBRs in rhodophytes and cryptophytes. A simplified scheme is shown for evolution of rhodophyte and cryptophyte algae and for acquisition of photosynthesis. Rhodophytes acquired photosynthesis via primary endosymbiosis of a cyanobacterium that had PcyA, PebA, and PebB (dotted line). Based on phylogenetic analysis (see Fig. 4 and Results), the ancestral rhodophyte retained all three enzymes. Most extant rhodophytes retain only PebA and PebB, but *C. merolae* instead retains PcyA. Cryptophytes acquired photosynthesis via secondary endosymbiosis of a rhodophyte (dashed line), giving rise to a complex plastid that retains a relict nucleus (nucleomorph) with a minimal eukaryotic genome. Extant cryptophyte FDBRs are closely related to rhodophyte FDBRs. We therefore propose that the initial secondary endosymbiosis by cryptophytes also transferred all three FDBRs to the ancestral cryptophyte. Most extant cryptophytes also retain only PebA and PebB, but PcyA is also present in some cryptophyte genera. LUCA, last universal cellular ancestor; LECA, last eukaryotic common ancestor.

Supporting Table S1. Identification of FDBR homologs in *Galdieria sulphuraria*[1]

Query	Accession	BLAST	Accession	HMMER	Accession
Cyanobacterial PcyA	WP_218080234	2e-14	EME26592	4.8e-15	EME26592
		9e-11	EME26797	3e-11	EME26797
Cyanobacterial PcyA	TE42_09505	2e-14	EME26592	1.6e-13	EME26592
		4e-07	EME26797	1e-07	EME26797
AX clade PcyA	BRADO1265	–		–	
AX clade PcyX	EBK42635	–		–	
Pre-PcyA	MBL9008304	–		–	
Pre-PcyA	CAP_1520	5e-11	EME26592	1.7e-10	EME26592
		1e-04	EME26797	0.005	EME26797
Pre-PcyA	POZ53557	–		0.0019	EME26592
Candidate pre-PcyA	HJK99203	4e-07	EME26592	3.4e-07	EME26592

Candidate pre-PcyA	MCS6914659	4e-06	EME26592	9.4e-05	EME26592
Candidate pre-PcyA	MBL9113084	–		0.041	EME26592
		–		0.063	EME26797
Candidate pre-PcyA	MCC7536511	–		–	
Candidate pre-PcyA	MCU0492826	4e-18	EME26592	2.1e-19	EME26592
		1e-13	EME26797	1.1e-15	EME26797
Candidate pre-PcyA	NDJ62509	1e-31	EME26592	3e-32	EME26592
		6e-23	EME26797	3.2e-24	EME26797
Candidate pre-PcyA	TNE48865	1e-16	EME26592	2.4e-18	EME26592
		1e-07	EME26797	3.3e-07	EME26797
Candidate pre-PcyA	TAF67438	3e-11	EME26797	1.9e-13	EME26592
		2e-10	EME26592	1.9e-12	EME26797
Cyanobacterial PebA	BAC89201	6e-68	EME26797	2.4e-64	EME26797
		6e-41	EME26592	1.7e-40	EME26592
Cyanobacterial PebB	BAC89202	7e-103	EME26592	1.5e-95	EME26592
		2e-30	EME26797	2.1e-31	EME26797
Unknown	D6734_12105	2e-13	EME26797	5.1e-14	EME26797
		6e-12	EME26592	1.1e-13	EME26592
Arabidopsis RCCR	3AGA_A	–		–	
Candidate CPO	MCX5884553	2e-08	EME30735	2.5e-06	EME30735

1. Searches under default significance thresholds for each of the indicated software packages using the indicated queries (all listed as searchable accessions in the NCBI Protein database). E-values are indicated, as are accessions for the *G. sulphuraria* sequences. EME26592 is annotated as a phycoerythrobilin:ferredoxin oxidoreductase (PEBB), EME26797 is annotated as a 15,16-dihydrobiliverdin:ferredoxin oxidoreductase (PEBA), and EME30735 is annotated as an oxygen-dependent coproporphyrinogen III oxidase (CPO).

Supporting Table S2. Comparison of phycobiliprotein lyases in *Galdieria sulphuraria* and *Cyanidioschyzon merolae*.

Lyase	Accession (NCBI)	% identity
CpcE	GJD06583.1 (*G. sulphuraria*)	38.28
	XP_005534793.1 (*C. merolae*)	
CpcF	XP_005702676.1 (*G. sulphuraria*)	35.17
	XP_005534794.1 (*C. merolae*)	
CpcS	GJD07013.1 (*G. sulphuraria*)	51.43
	XP_005538397.1 (*C. merolae*)	
CpcU	YP_009051127.1 (*G. sulphuraria*)	/
CpcT	XP_005709309.1 (*G. sulphuraria*)	44.66
	XP_005536644.1 (*C. merolae*)	

Supporting Table S3. *Galdieria sulphuraria* growth media and Trace elements composition.

Component	Concentration
$CaCl_2$ x 2 H_2O	0.14 mM
FeNa-EDTA	0.04 mM
KH_2PO_4 x 2 H_2O	2.2 mM
$MgSO_4$	1.22 mM
NaCl	0.34 mM
$(NH_4)_2SO_4$ x 7 H_2O	11.35 mM
Glucose	25 mM
Trace elements	2 mL/L

Trace elements	Concentration
$CoCl_2$ x 6 H_2O	0.17 mM
$CuSO_4$ x 5 H_2O	0.32 mM
H_3BO_3	46.26 mM
$MnCl_2$ x 4 H_2O	9.2 mM
$NaVO_3$ x 4 H_2O	0.21 mM
$(NH_4)_6Mo_7O_{24}$ x 4 H_2O	0.85 mM
$ZnSO_4$ x 7 H_2O	7.7 mM
